# Metabolomics reveal alterations in arachidonic acid metabolism in *Schistosoma mekongi* after exposure to praziquantel

**DOI:** 10.1371/journal.pntd.0009706

**Published:** 2021-09-02

**Authors:** Peerut Chienwichai, Phornpimon Tipthara, Joel Tarning, Yanin Limpanont, Phiraphol Chusongsang, Yupa Chusongsang, Poom Adisakwattana, Onrapak Reamtong

**Affiliations:** 1 Faculty of Medicine and Public Health, HRH Princess Chulabhorn College of Medical Science, Chulabhorn Royal Academy, Bangkok, Thailand; 2 Mahidol Oxford Tropical Medicine Research Unit, Faculty of Tropical Medicine, Mahidol University, Bangkok, Thailand; 3 Centre for Tropical Medicine and Global Health, Nuffield Department of Clinical Medicine, University of Oxford, Oxford, United Kingdom; 4 Department of Social and Environmental Medicine, Faculty of Tropical Medicine, Mahidol University, Bangkok, Thailand; 5 Department of Helminthology, Faculty of Tropical Medicine, Mahidol University, Bangkok, Thailand; 6 Department of Molecular Tropical Medicine and Genetics, Faculty of Tropical Medicine, Mahidol University, Bangkok, Thailand; National University of Ireland Galway, IRELAND

## Abstract

**Background:**

Mekong schistosomiasis is a parasitic disease caused by the blood-dwelling fluke *Schistosoma mekongi*. This disease contributes to human morbidity and mortality in the Mekong region, posing a public health threat to people in the area. Currently, praziquantel (PZQ) is the drug of choice for the treatment of Mekong schistosomiasis. However, the molecular mechanisms of PZQ action remain unclear, and *Schistosoma* PZQ resistance has been reported occasionally. Through this research, we aimed to use a metabolomic approach to identify the potentially altered metabolic pathways in *S*. *mekongi* associated with PZQ treatment.

**Methodology/Principal findings:**

Adult stage *S*. *mekongi* were treated with 0, 20, 40, or 100 μg/mL PZQ *in vitro*. After an hour of exposure to PZQ, schistosome metabolites were extracted and studied with mass spectrometry. The metabolomic data for the treatment groups were analyzed with the XCMS online platform and compared with data for the no treatment group. After low, medium (IC_50_), and high doses of PZQ, we found changes in 1,007 metabolites, of which phosphatidylserine and anandamide were the major differential metabolites by multivariate and pairwise analysis. In the pathway analysis, arachidonic acid metabolism was found to be altered following PZQ treatment, indicating that this pathway may be affected by the drug and potentially considered as a novel target for anti-schistosomiasis drug development.

**Conclusions/Significance:**

Our findings suggest that arachidonic acid metabolism is a possible target in the parasiticidal effects of PZQ against *S*. *mekongi*. Identifying potential targets of the effective drug PZQ provides an interesting viewpoint for the discovery and development of new agents that could enhance the prevention and treatment of schistosomiasis.

## Author summary

Schistosomiasis is a major neglected tropical disease that is a public health threat in many countries worldwide. Hundreds of millions of people live in endemic areas and are at risk for this disease. PZQ is the drug of choice for many parasitic infections and is the primary drug used to treat schistosomiasis. PZQ resistance is a problem in endemic areas, emphasizing the need for novel drugs in the fight against schistosomiasis. This study aimed to elucidate the molecular mechanisms of how PZQ affects schistosomes and thereby identify novel targets for anthelminthic drug development. Our findings highlighted the anandamide and arachidonic acid metabolism pathways as important targets of the anti-schistosomal effects of PZQ. These pathways might present valid targets for drug development for the treatment of schistosomiasis.

## Introduction

Schistosomiasis, or bilharzia, is a disease caused by blood-dwelling flukes of the genus *Schistosoma*. There are six species of *Schistosoma* fluke that infect humans: *S*. *mansoni*, *S*. *japonicum*, *S*. *intercalatum*, *S*. *guineensis*, *S*. *mekongi* (intestinal schistosomiasis), and *S*. *haematobium* (urogenital schistosomiasis) [1]. The disease affects more than 250 million people worldwide and mortality cases reach 280,000 per year in the Sub-Saharan region of Africa alone [2]. Host immune responses to parasite eggs cause abdominal pain, diarrhea, bloody stool, liver enlargement (for intestinal schistosomiasis), pelvic pain, hematuria, and dysuria (for urogenital schistosomiasis) [1,2], and patients may develop advanced symptoms leading to disability and death [1,2].

To date, the prevention and treatment of *Schistosoma* spp. infections have relied on only one drug: praziquantel (PZQ). The anthelminthic PZQ has been used to control parasitic infections since 1972 [3] and shows excellent efficacy against many species of cestodes and trematodes, including *Schistosoma spp*. [3,4]. Although PZQ has been extensively used for decades, the molecular targets of PZQ and its effects on parasite metabolism remain unclear [5–8]. Several studies have attempted to understand the drug’s effects in schistosomes. After flukes were exposed to PZQ, their movement was halted by muscular paralysis; moreover, vacuolization and blebbing were observed on their outer surface, indicating damage to the tegument layer [9]. The induction of intracellular calcium influx is the most recognized mechanism of action for PZQ [5–7,9–11], and PZQ has been hypothesized to interfere with the interactions between the α and β subunits of voltage-gated calcium channels, leading to increased calcium uptake by myocytes. High levels of intracellular calcium cause sustained muscular contraction, resulting in spastic paralysis [6,11]. However, much remains unknown about the mode of action of PZQ. Furthermore, low susceptibility and resistance to PZQ have been reported in many regions. It has been reported that some populations of *S*. *mansoni* [12,13], *S*. *japonicum* [14], *S*. *hematobium* [15], and other cestode species [16,17] can survive PZQ treatment at the current therapeutic dose, which is a worrying indication that PZQ might become ineffective in the near future.

Metabolomics, which is the global analysis of small molecule metabolites found in living organisms under certain conditions [18,19], is a powerful tool for identifying novel drug targets, biomarker discovery, the monitoring of disease, and studying disease pathogenesis, for example. [20,21]. Metabolomic approaches have been widely applied in the search for new treatment targets for parasitic infections. For example, Schalkwijk *et al*. found that the coenzyme A biosynthesis pathway of *Plasmodium falciparum* was vulnerable to pantothenamide treatment, which can thus be used as an antimalarial agent. Pantothenamide inhibited acetyl-CoA synthesis by acting as a coenzyme A analog and can kill the malarial parasite effectively [22]. Hennig *et al*. studied the metabolomic profiles of the intracellular amastigote stage of *Trypanosoma cruzi* treated with six drugs and found that the tricarboxylic acid cycle was the most prominently affected pathway [18]. In the development of drugs against *Schistosoma spp*., metabolomics has only been applied to study perhexiline maleate [19], and no data are available on the metabolic changes after PZQ treatment in this schistosomal parasite. Therefore, using metabolomic methodology and in-depth pathway analysis may improve our understanding of PZQ modes of action. Furthermore, studying PZQ-related pathways may lead to the identification of novel targets for the development of further anthelminthic drugs. In this study, we explored the alterations in the profile of metabolites of *S*. *mekongi* adult worms after PZQ exposure at low, medium, and high doses. The differential metabolites were subjected to pathway analysis to get insights on the mechanisms of action, and to highlight potential PZQ targets in the treatment of Mekongi schistosomes. This information could also shed some light on resistance development and pinpoint important candidate metabolites for future drug development.

## Methods

### Ethics statement

Procedures involving animals were performed in accordance with the guidelines for the use of animals at the National Research Council of Thailand (NRCT) and were approved by Faculty of Tropical Medicine Animal Care and Use Committee (FTM-ACUC), Mahidol University (Approval number: FTM-ACUC 032–2020).

### Life cycle of *S*. *mekongi* and PZQ treatment

For *S*. *mekongi* culture, freshwater snails (*Neotricula aperta*) were used as intermediate hosts, and mice (*Mus musculus*) were used as definitive hosts, as previously described [23]. The snails were collected from their natural habitat in the Mekong River and tributaries in Thailand and maintained at the Applied Malacology Laboratory, Department of Social and Environmental Medicine, Faculty of Tropical Medicine, Mahidol University, Bangkok, Thailand. The natural infection of trematodes were checked by light shedding method. Similarly, 8-week-old female ICR mice were purchased from the National Laboratory Animal Center, Mahidol University. Ten miracidia per snail were used to infect 300–400 snails and the cercaria from at least 50 snails were used for a mouse infection. Twenty-four mice were infected with 25–30 cercariae per mouse percutaneously and housed in controlled conditions at the Animal Care Unit, Faculty of Tropical Medicine, Mahidol University. Eight weeks following infection, adult worms were collected by hepatic perfusion with sterile 0.85% saline solution.

Adult *S*. *mekongi* obtained by hepatic perfusion of mice were cultured in RPMI medium (Hyclone, GE Healthcare, Little Chalfont, UK) in a humidified 5% CO_2_ incubator at 37°C. Thereafter, PZQ (Tokyo Chemical Industry, Tokyo, Japan) was dissolved in dimethyl sulfoxide and diluted to a final concentration of 0 (control), 20, 40 (IC_50_), and 100 μg/mL with RPMI medium. The inhibitory concentration associated with 50% effect (IC_50_), used in this study, was determined as described in our previous publication [24]. Each concentration of PZQ was added to 10 pairs of *S*. *mekongi* and three biological replicates were used for each concentration with several batches of worms. Worm movement under a microscope was used as an indicator for viability. At the end of one hour exposure, worms were picked to see whether they moved or not. Number of dead worms were recorded and compared between PZQ doses. All worms were collected and kept at −80°C until metabolite extraction was performed.

### Metabolite extraction

Metabolite extraction was performed according to a previously described study [25]. All worms from each condition were transferred into 1.5-mL microcentrifuge tubes and homogenized in 500 μL methanol. At which point, the tubes were snap-frozen in liquid nitrogen and thawed prior to centrifuging at 800 × *g* for 1 min at 4°C. The supernatant was collected and placed in a new tube, and the pellet was extracted again with the same protocol. Following centrifugation, the supernatant from the second extraction was pooled into a tube containing the supernatant from the first extraction. The pellet was resuspended in 250 μL of deionized H_2_O before snap-freezing in liquid nitrogen and thawing. The supernatant was obtained by centrifugation at 15,000 × *g* for 1 min at 4°C and then pooled with the previous tube. The tubes containing the pooled supernatants were centrifuged at 15,000 × *g* for 1 min at 4°C to remove the remaining debris. The clear supernatant was transferred to a new tube and later dried in a speed vacuum (Tomy Digital Biology, Tokyo, Japan).

### Metabolite identification by mass spectrometry

The ultra-high performance liquid chromatography (UHPLC; Agilent 1260 Quaternary pump, Agilent 1260 High Performance Autosampler, and Agilent 1290 Thermostatted Column Compartment SL, Agilent Technologies) coupled to a quadrupole time-of-flight mass spectrometer (Q-TOF-MS) (TripleTOF 5600^+^, SCIEX, US) with electrospray ionization (ESI) using a DuoSpray ion source. The mobile phase system for UHPLC separation was water containing 0.1% formic acid (mobile phase A) and acetonitrile containing 0.1% formic acid (mobile phase B). The metabolite pellet was reconstituted in 200 μL of mobile phase A:B at a ratio of 50:50 (vol/vol) and transferred to a liquid chromatography (LC) vial for injection. LC vials were kept in the auto-sampler at 6°C during the analysis. Five microliters of sample was injected onto a C18 reversed phase column (ACQUITY UPLC HSST3, 2.1 × 100 mm, 1.8 μM, Waters) protected by a pre-column (ACQUITY UPLC HSST3, 2.1 × 5 mm, 1.8 μM, Waters) for separation by UHPLC at a flow rate of 0.3 mL/min at 40°C. The UHPLC elution gradient was started at 5% mobile phase B for 2.0 min (0.0–2.0 min), 5%–60% B for 0.5 min (2.0–2.5 min), 60%–80% B for 1.5 min (2.5–4.0 min), 80%–100% B for 8.0 min (4.0–12.0 min), 100% B for 5 min (12.0–17.0 min), 100%–5% B for 0.1 min (17.0–17.1 min), and 5% B for 2.9 min (17.1–20.0 min). The UHPLC-Q-TOF-MS system, mass ion chromatogram, and mass spectra were acquired by Analyst Software version 1.7 (SCIEX). The Q-TOF-MS was operated in positive (+ESI) and negative (-ESI) electrospray ionization modes. Ion source gas 1 was set at 45 psi, ion source gas 2 at 40 psi, curtain gas at 30 psi, and source temperature at 450°C. Ion spray voltage floating was set at 4500 V in positive mode and at -4500 V in negative mode. The de-clustering potential was set to 100 V in positive mode and to −100 V in negative mode. Data were acquired in the informative dependent acquisition mode composed of a TOF-MS scan, and 10 dependent product ion scans were used in the high sensitivity mode with dynamic background subtraction. The collision energy was set to 30 V, and the collision energy spread was set to 15 V. The mass range of the TOF-MS scan was m/z 100–1,000, and the product ion scan was set to m/z 50−1,000. Equal aliquots of each metabolite sample were pooled to form the quality control (QC) samples. The QC samples were injected before, during, and after sample analysis to assess the system performance. Raw mass spectra files (.wiff) were processed and visualized using the XCMS Version 3.7.1 online tool (The Scripps Research Institute, CA, USA), and metabolites were identified using METLIN (The Scripps Research Institute) as a database [26]. All metabolites with 95% confidence were reported in this paper.

### Data analysis and pathway enrichment

Comparisons between the control sample (0 μg/mL PZQ treatment) and other samples were performed with “Pairwise” mode, while multivariate analysis of all samples was also performed using “Multigroup” mode. The parameters for identification of metabolites were chosen according to “UPLC/Triple TOF pos” protocol. In brief, the protocol composed of 5 parameters, including feature extraction, alignment, statistics, annotation, and identification. For feature extraction, parameters composed of positive polarity, 15 ppm maximal tolerated m/z deviation, 5–20 seconds peak width, 6 signal/noise threshold, and 0.01 minimum difference in m/z. For alignment, parameters composed of 5 seconds allowable retention time duration, 0.5 minimum fraction, and 0.015 width of overlapping m/z. For statistics, unpaired parametric t-test (Welch t-test) was used for pairwise comparison and Kruskal-Wallis non-parametric test was used for multiple group comparison. Metabolites showing a 1.5-fold difference with a *p-*value of less than 0.01 were identified as significantly different. For Annotation, parameters composed of 5 ppm error, 0.01 m/z absolute error, and search for isotopic features and their adduct formations. For identification, 74 common adducts were considered for database search with 5 ppm tolerance for database search. Principal component analysis (PCA) plot and volcano plot were used for further analysis of metabolomic data. For PCA, 1000 modify loadings threshold, pareto scaling option, and center were selected for generating the plot. For volcano plot, Log_2_ of fold change and -Log of *p*-value were calculated for generating the plot. The top 20 metabolites with lowest *p-*values and highest fold change from multivariate analysis were presented in table, while the top 10 metabolites with the lowest *p-*values and highest fold changes were labeled in the plots and table. Pathway analysis was performed using the “Activity network” feature of XCMS. These prominent pathways were depicted based on the Kyoto Encyclopedia of Genes and Genomes (KEGG) pathway database [27–29].

### Protein sequence alignment

Fatty acid amide hydrolase protein sequence of *S*. *mekongi* was retrieved from an in-house transcriptome database of our previous study [23]. Protein sequences of other organisms in this study were retrieved from the non-redundance protein sequence database of the National Center for Biotechnology Information (NCBI) and UniProt Knowledgebase (UniprotKB). All sequences were used to perform alignments, which their phylogenetic tree and percentage identity were evaluated using Clustal Omega software. For the parameter setting, number of combined iterations, max guide tree iterations, max HMM iteration were set as 0. The MBED-LIKE clustering guide-tree and MBED-LIKE clustering iteration were used.

## Results

### Alteration of *S*. *mekongi* metabolomes after PZQ treatment

**Fig 1:**
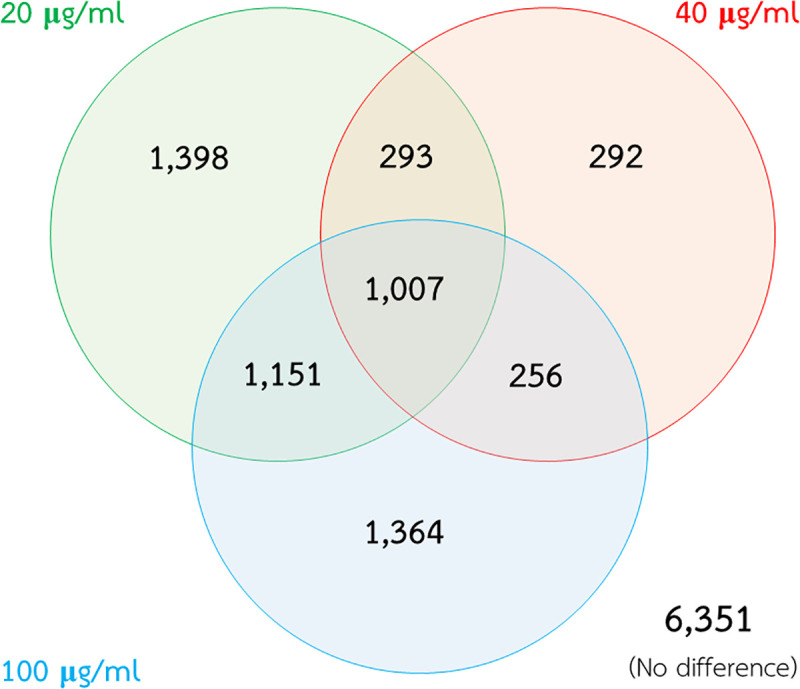
Alteration of *S*. *mekongi* metabolites after PZQ treatment. Green, red, and blue circles represent differential metabolites after low-, medium-, and high-dose PZQ treatment, respectively.

To elucidate the effects of PZQ on the *S*. *mekongi* metabolome, worms were exposed to low (20 μg/mL), medium (40 μg/mL), and high (100 μg/mL) PZQ concentrations. In our previous study, we exposed the three concentrations of PZQ to adult worms for an hour and worm movement were used as indicator of viability. At the end of treatment duration, worms those did not response to agitation were considered as death. The PZQ treated worm at high dose were observed the complete sprawling and stretching out of the body. While, the controls were noticed the body-bend amplitude during locomotion. We found that the three concentrations reduced the viability of parasites by 0%, 46.7%, and 100%, respectively [24]. Metabolites from these three conditions were extracted and compared with those from the control (0 μg/mL PZQ). A total of 12,112 metabolites were identified by mass spectrometry, of which 6,351 remained unchanged and 5,761 were altered after PZQ exposure. Low-, medium-, and high-dose PZQ treatment caused alterations in 3,849, 1,848, and 3,778 metabolites, respectively. There were 1,007 altered metabolites shared among all three conditions (Fig 1).

**Fig 2:**
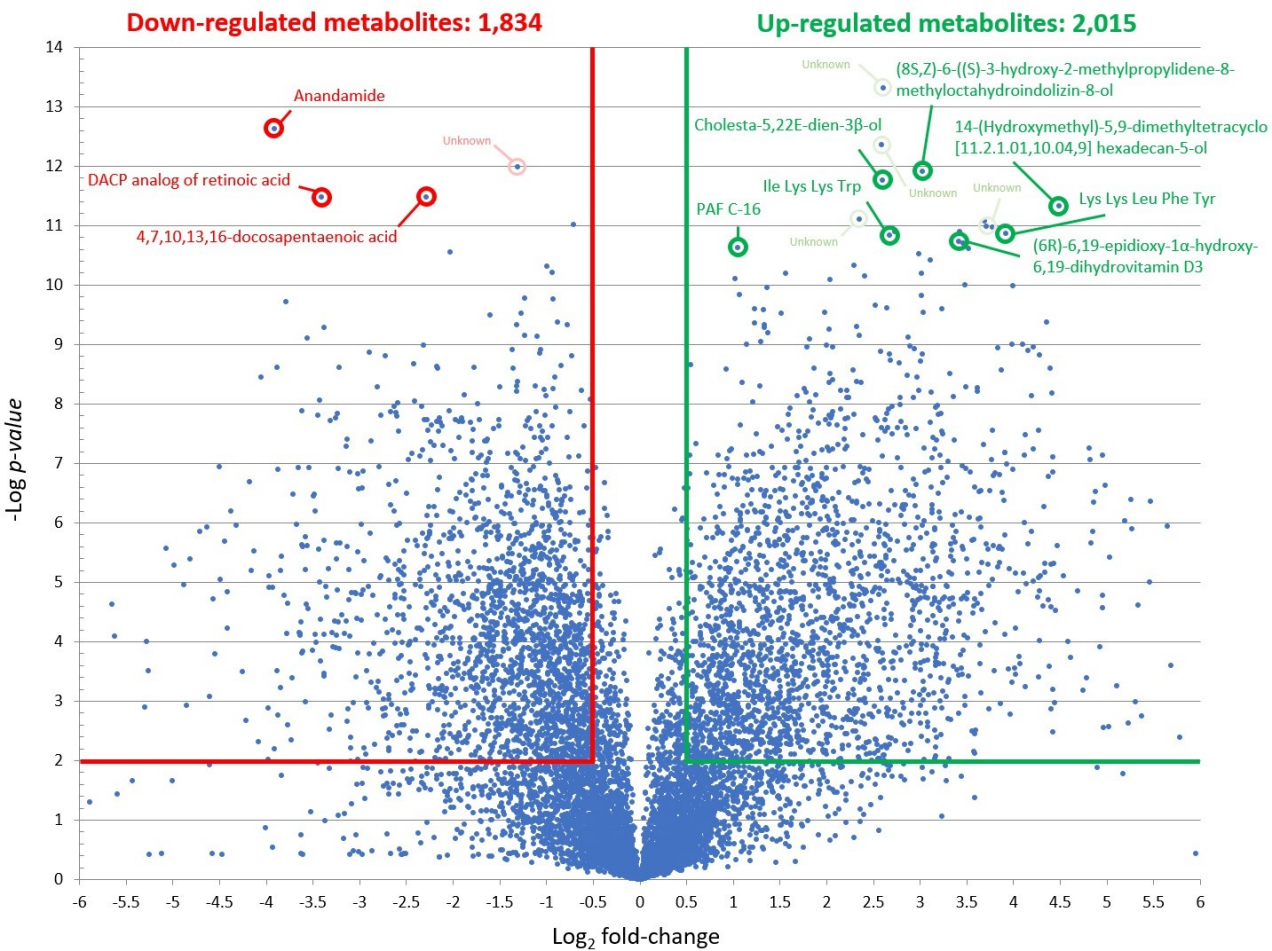
Volcano plots showing differentially expressed metabolites of *S*. *mekongi* following low-dose PZQ treatment. Horizontal green and red lines represent *p-*values equal to 0.01. Vertical green and red lines represent fold changes equal to 1.5 and −1.5, respectively. Top 10 metabolites with the lowest *p*-values (highly significant) and largest fold changes of each dose were labeled. Green and red refer to higher and lower abundance of metabolites, respectively.

**Fig 3:**
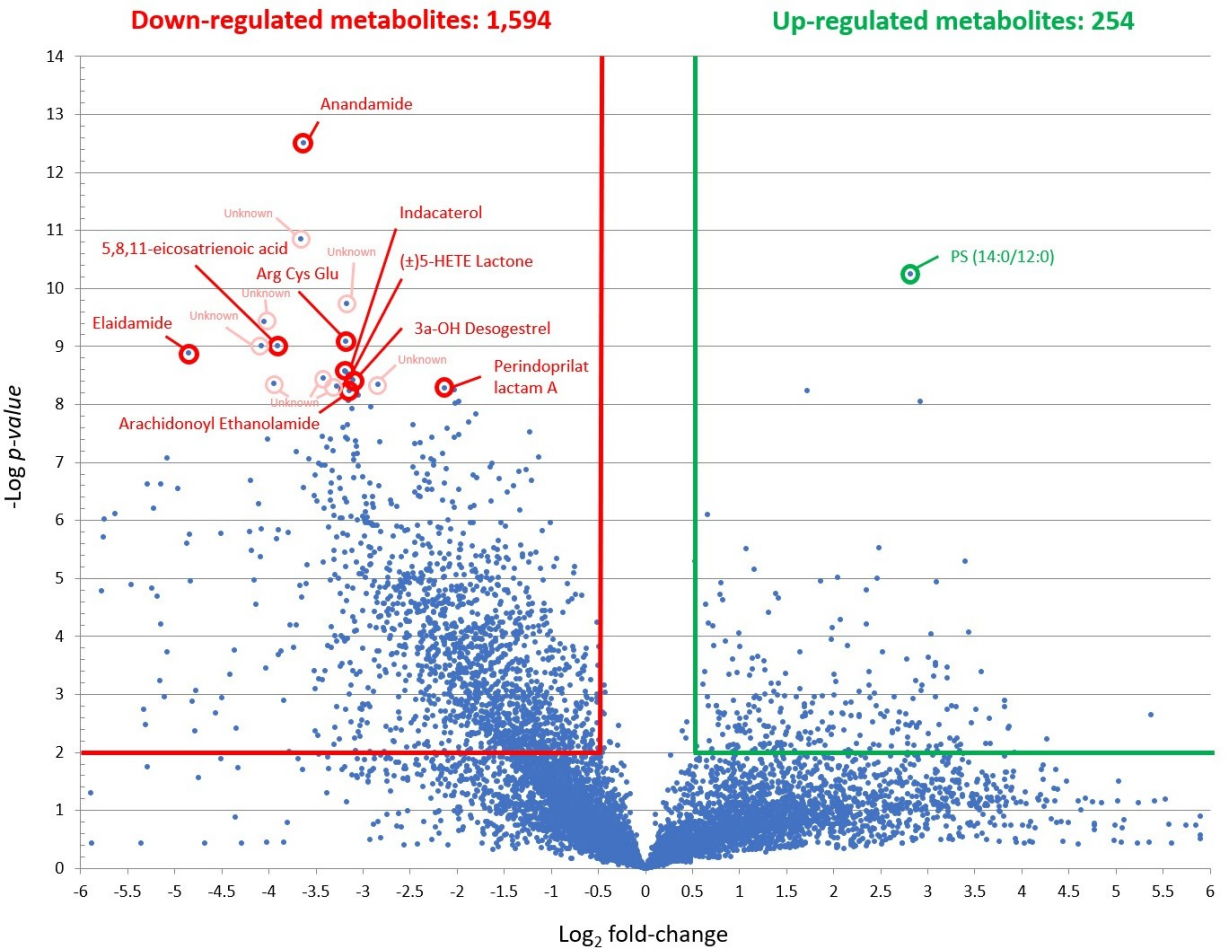
Volcano plots showing differentially expressed metabolites of *S*. *mekongi* following medium-dose PZQ treatment. Horizontal green and red lines represent *p-*values equal to 0.01. Vertical green and red lines represent fold changes equal to 1.5 and −1.5, respectively. Top 10 metabolites with the lowest *p*-values (highly significant) and largest fold changes of each dose were labeled. Green and red refer to higher and lower abundance of metabolites, respectively.

**Fig 4:**
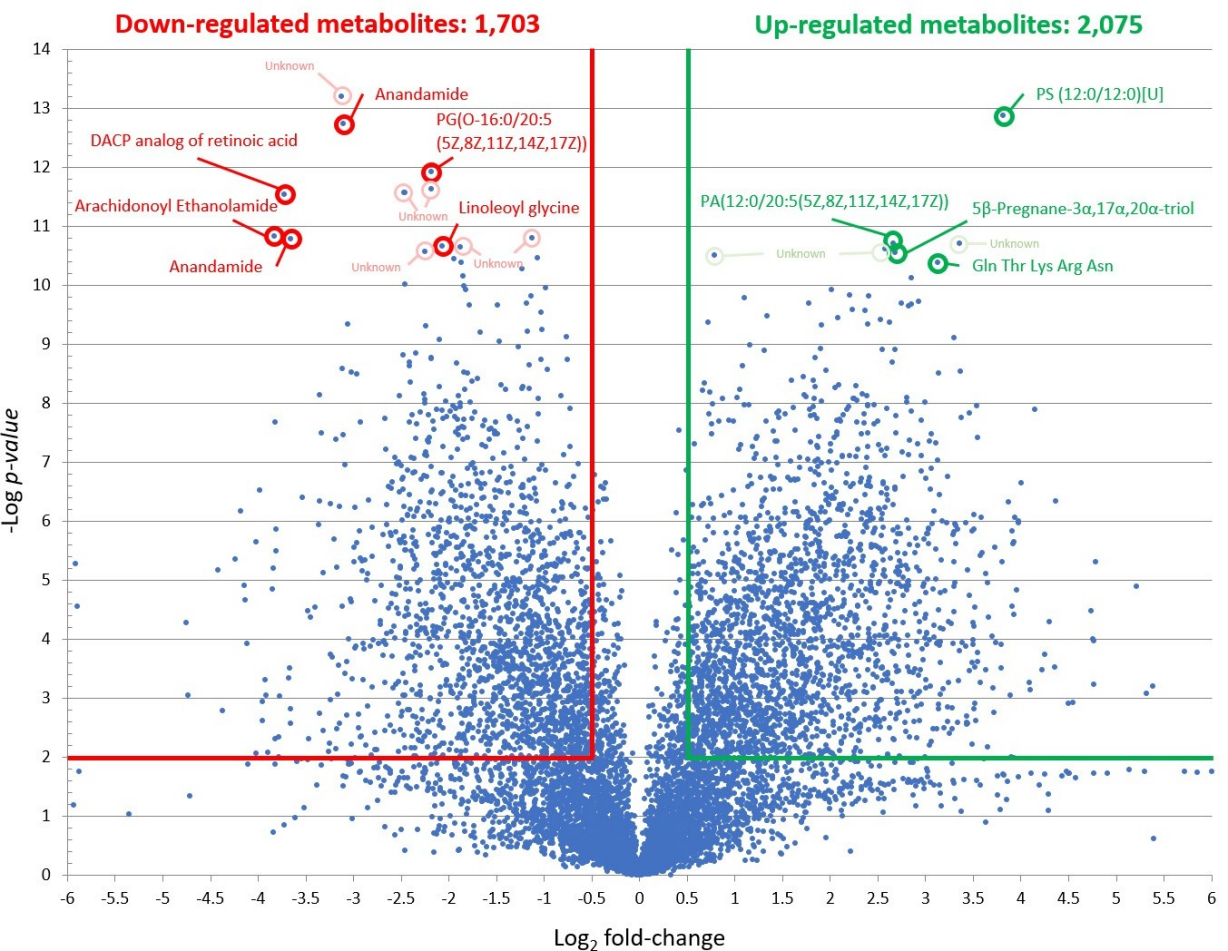
Volcano plots showing differentially expressed metabolites of *S*. *mekongi* following high-dose PZQ treatment. Horizontal green and red lines represent *p-*values equal to 0.01. Vertical green and red lines represent fold changes equal to 1.5 and −1.5, respectively. Top 10 metabolites with the lowest *p*-values (highly significant) and largest fold changes of each dose were labeled. Green and red refer to higher and lower abundance of metabolites, respectively.

**Fig 5:**
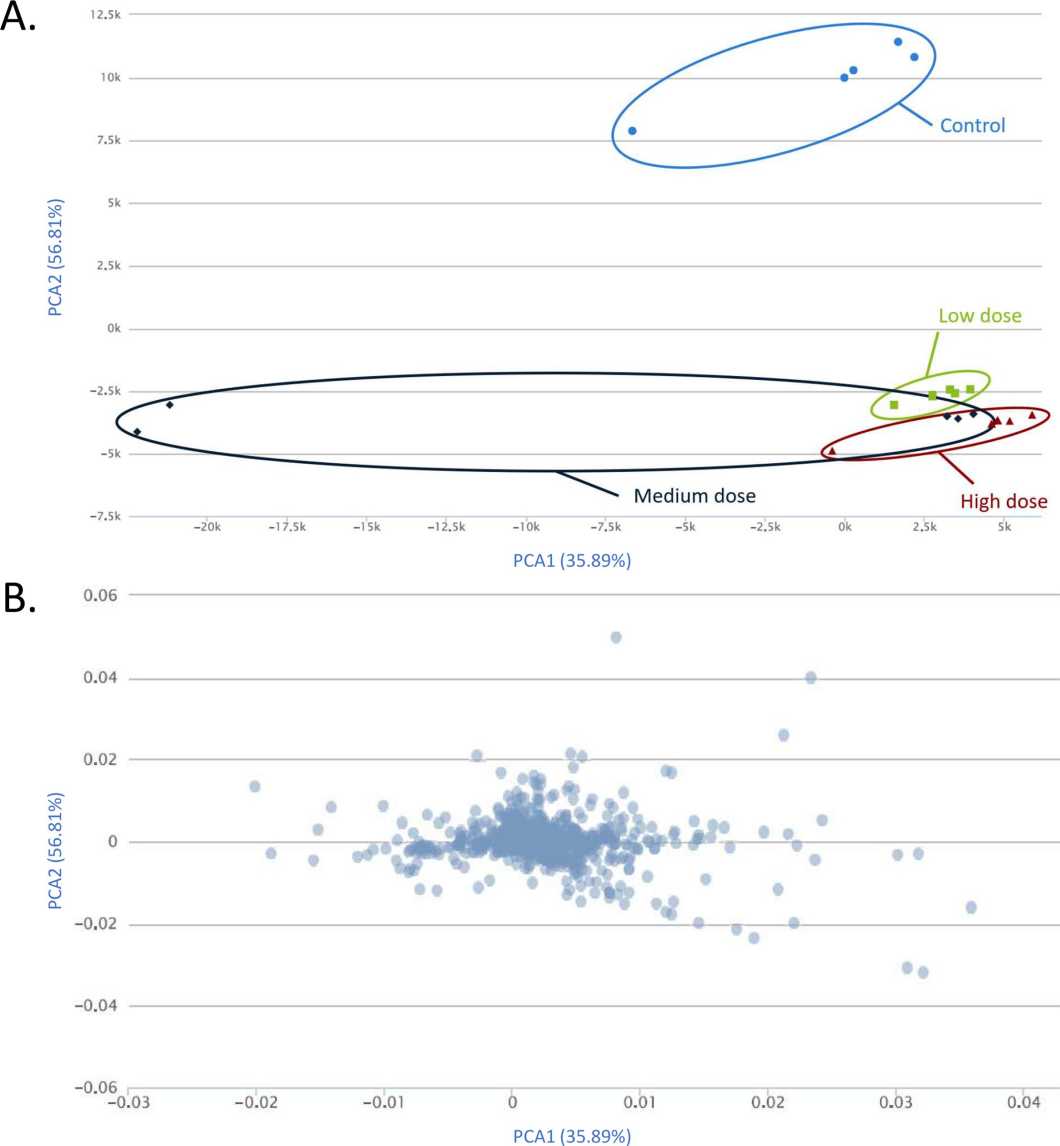
Principal component analysis (PCA) plot of metabolomic data from control, low-, medium-, and high-dose PZQ treatment. (A) PCA score plot. Blue circles represent dataset of control group (Centroid = -506.301, 10080.555). Green squares represent dataset of low-dose PZQ treatment group (Centroid = 3011.11, -2643.879). Black diamonds represent dataset of medium-dose PZQ treatment group (Centroid = -6516.275, -3540.422). Scarlet triangles represent dataset of high-dose PZQ treatment group (Centroid = 4011.466, -3896.345). (B) PCA loading plot.

The three PZQ concentrations resulted in different levels of *S*. *mekongi* metabolite alteration. At the low dose, there were 2,015 and 1,834 metabolites those their level increased and decreased from the treatment, respectively (Fig 2). While the medium dose caused 254 metabolites to be increased and 1,594 metabolites to be decreased (Fig 3). The high-dose PZQ led to 2,075 higher and 1,703 lower level of metabolites (Fig 4). With multivariate analysis, abundance of 4,198 metabolites were altered after PZQ treatment. Principal component analysis (PCA) plot demonstrated the distinct separation of principal components of treatment groups from control group, indicating effects of PZQ on metabolite levels of *S*. *mekongi* (Fig 5).

**Fig 6:**
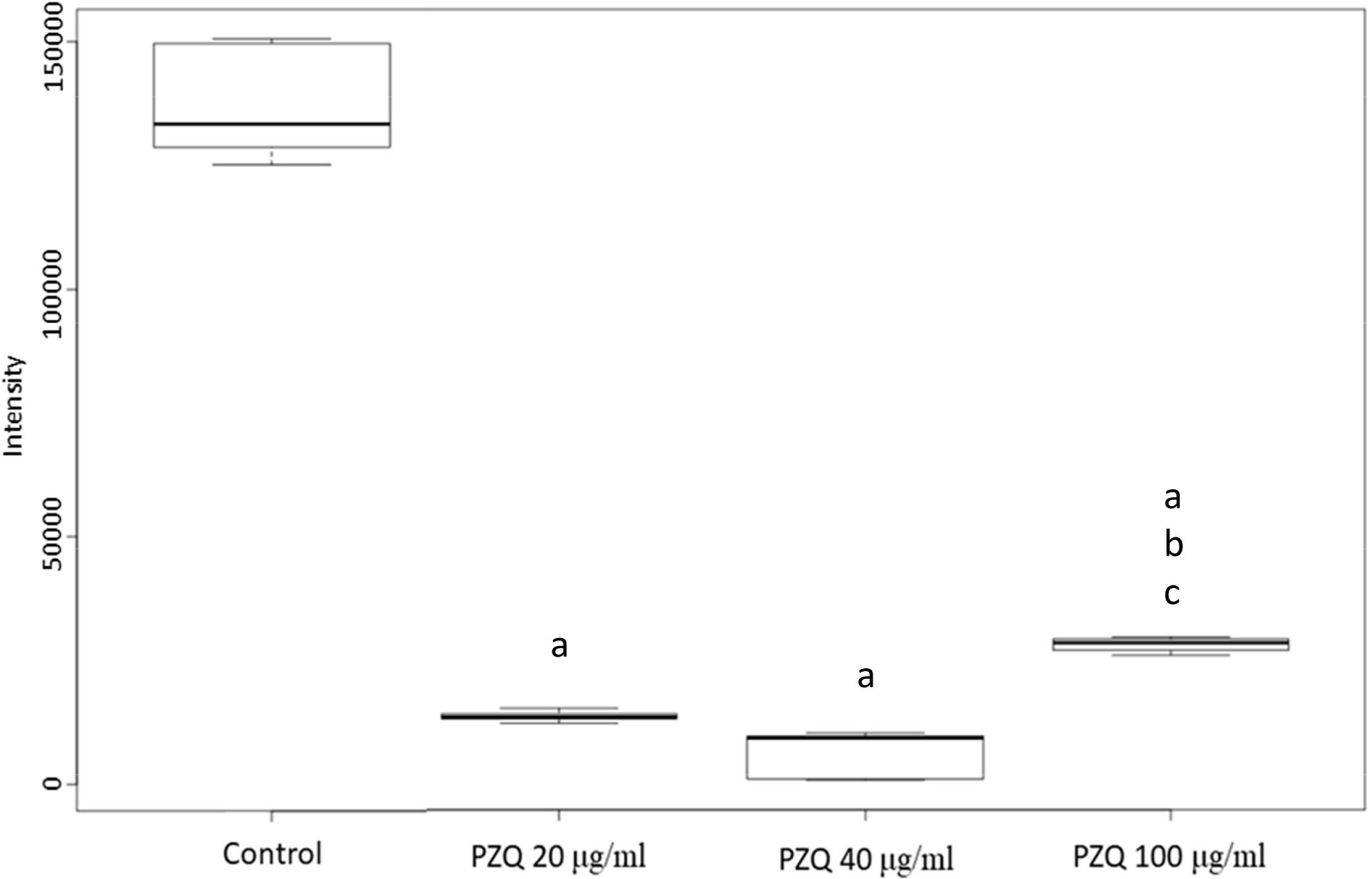
Increased level of PS (14:0/12:0) after different doses of PZQ treatment of *S*. *mekongi*. Boxplot shows level of PS (14:0/12:0) following different doses of PZQ. “a” indicates statistical differences at *p* < 0.01 from control group. “b” indicates statistical differences at *p* < 0.01 from low dose group. “c” indicates statistical differences at *p* < 0.01 from medium dose group.

**Fig 7:**
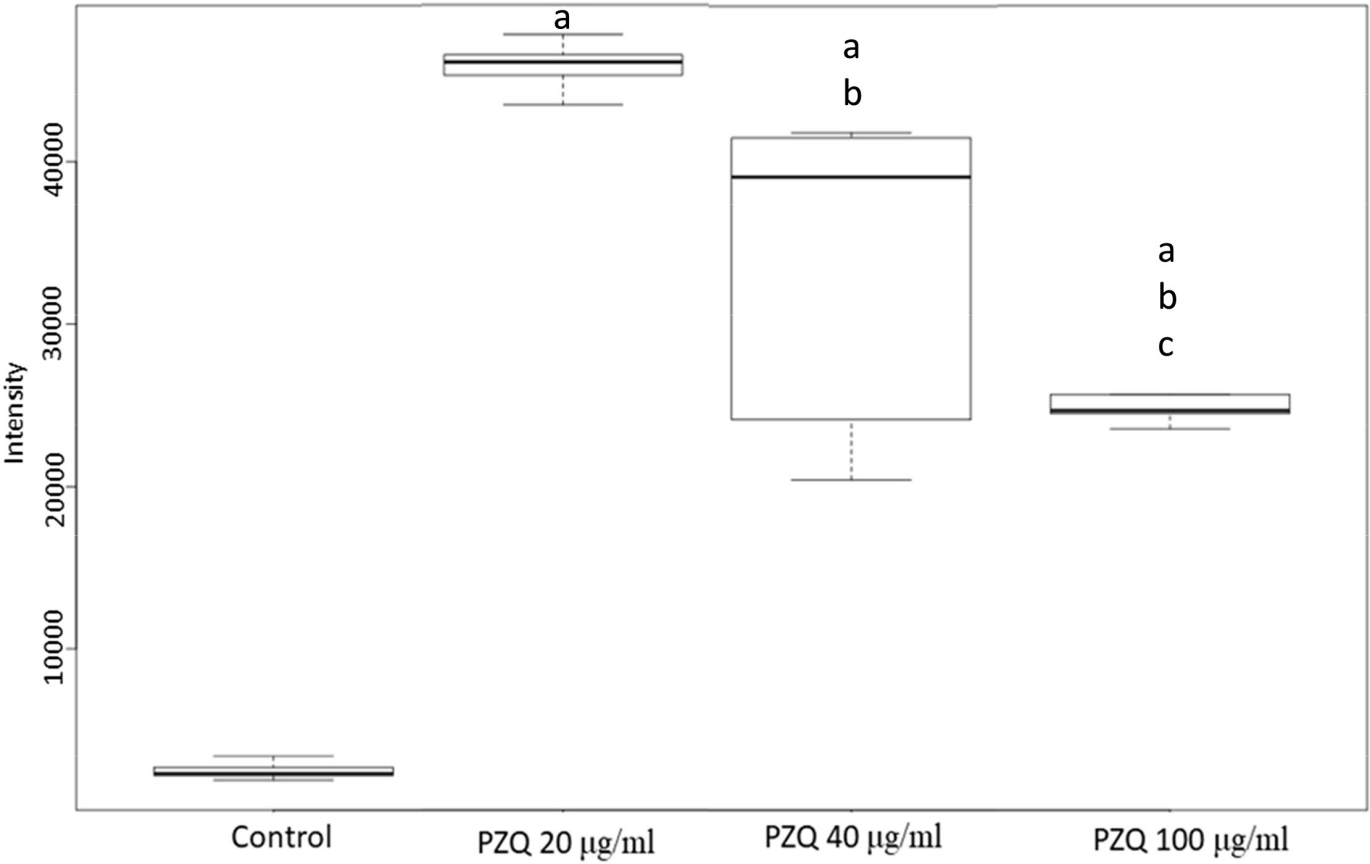
Decreased level of anandamide after different doses of PZQ treatment of *S*. *mekongi*. Boxplot showing levels of anandamide following different doses of PZQ. “a” indicate statistical difference at *p* < 0.01. “b” indicates statistical differences at *p* < 0.01 from low dose group. “c” indicates statistical differences at *p* < 0.01 from medium dose group.

The top-20 metabolites with significant increase and decrease in their abundance are shown in Tables 1 and 2, respectively. In addition, top-10 metabolites of each condition with increased and decreased level are shown in S1 and S2 Tables, respectively. Among all differential metabolites, a group of phosphatidylserine (PS) was uniquely increased following PZQ treatment. Level of PS (12:0/13:0) (METLIN ID: 3870) and PS (12:0/16:1(9Z)) (METLIN ID: 77713) were significantly increased by multivariate analysis (Table 1) and level of PS (14:0/12:0) (METLIN ID: 78595) was increased in all conditions (Fig 6 and S1 Table). Whereas anandamide (20:5, n-3) (METLIN ID: 36743) was the only metabolite showing lower level in all conditions (Fig 7 and S2 Table). Multivariate analysis revealed consistent findings that level of anandamide (20:5, n-3) and anandamide (18:3, n-6) (METLIN ID: 36739) were significantly decreased (Table 2). Anandamide is an intracellular ligand that can bind to the endocannabinoid receptor and is involved in many signal transductions [30], while PS (14:0/12:0) is localized on the parasite’s surface and plays key roles in cell-cycle signaling and apoptosis [10].

**Table 1:** Top 20 metabolites of *S*. *mekongi* with increased level after PZQ treatment by multivariate analysis.

No.	Chemical formula	Exact mass	Ion adduct	Mass error (ppm)	Fold change			*p*-*value*	Potential metabolite	METLIN ID
					Low dose	Medium dose	High dose			
1	C_45_H_62_O_4_	689.4572	[M+Na]+	5	6.3	10.4	2.8	0.00047	Trans-Geranylgeranylbixin	89894
2	C_20_H_32_O_3_	321.2425	[M+H]+	0	3.1	2.4	1.9	0.00047	20-hydroxy-5Z,8Z,11Z,14Z-eicosatetraenoic acid	35316
3	C_20_H_34_O_3_	323.2583	[M+H]+	1	7.7	3.0	3.2	0.00054	5S-hydroxy-6E,8Z,11Z-eicosatrienoic acid	36267
4	C_35_H_64_NO_8_P	680.4235	[M+K]+[M+H]+ [M+H-C_2_H_4_]+	4	3.6	4.8	2.4	0.00054	1-dodecanoyl-2-(6Z,9Z,12Z-octadecatrienoyl)-glycero-3-phosphoethanolamine (PE(12:0/18:3(6Z,9Z,12Z)))	76602
5	C_18_H_28_O_3_	293.2115	[M+H]+	1	3.3	9.4	2.8	0.00060	Alpha-licanic acid	35858
6	C_18_H_32_O_2_	281.2478	[M+H-NH_3_]+	1	2.5	7.9	1.6	0.00060	5Z,12Z-otadecadienoic acid	34788
7	C_27_H_44_O_3_	439.3172	[M+Na]+	2	6.6	1.7	3.1	0.00060	Dormatinone	41664
8	C_19_H_32_O_3_	331.2245	[M+Na]+	1	7.8	2.4	4.6	0.00061	Methyl 9,10-epoxy-12,15-octadecadienoate	74809
9	C_27_H_42_O	383.3303	[M+H]+	1	3.5	2.2	5.3	0.00061	Cholesta-4,6-dien-3-one	41650
10	C_19_H_32_O_2_	293.2478	[M+H]+	1	22.5	8.3	15.6	0.00067	14-(Hydroxymethyl)-5,9-dimethyltetracyclo [11.2.1.01,10.04,9]hexadecan-5-ol	984928
11	C_31_H_60_NO_10_P	638.4009	[M+H]+	3	5.9	24.7	3.3	0.00067	1-dodecanoyl-2-tridecanoyl-sn-glycero-3-phosphoserine (PS(12:0/13:0))	3870
12	C_24_H_40_O_3_	399.2868	[M+Na]+	0	6.1	2.4	5.4	0.00067	6α-Hydroxy-5β-cholan-24-oic Acid	42624
13	C_22_H_41_NO_4_	384.3101	[M+H]+	2	2.4	1.6	2.4	0.00074	N-oleoyl threonine	35490
14	C_24_H_47_NO_3_	398.3630	[M+K]+[M+Na]+[M+H]+ [M+H-NH_3_]+	0	7.3	4.9	11.9	0.00074	C-6 Ceramide	63012
15	C_34_H_64_NO_10_P	678.4331	[M+Na]+	1	2.9	3.9	3.9	0.00074	1-dodecanoyl-2-(9Z-hexadecenoyl)-glycero-3-phosphoserine (PS(12:0/16:1(9Z)))	77713
16	C_27_H_44_O_3_	417.3362	[M+H-COCH_2_]+	0	2.7	4.1	2.5	0.00074	Dormatinone	41664
17	C_27_H_42_O	383.3311	[M+H-H_2_O]+	1	3.5	2.1	3.7	0.00078	Zymosterone	57607
18	C_36_H_55_N_7_O_7_	698.4224	[M+K]+ M+Na]+ [M+H]+	2	15.2	7.6	8.7	0.00081	Lys Lys Leu Phe Tyr	264578
19	C_27_H_47_NO_2_	418.3675	[M+H+NH_3_]+	1	5.7	3.0	9.4	0.00081	N-(2R-methyl-3-hydroxy-ethyl)-16,16-dimethyl-5Z,8Z,11Z,14Z-docosatetraenoyl amine	36712
20	C_27_H_44_O	385.3467	[M+H]+	0	2.1	9.4	5.7	0.00089	Cholesta-5,22E-dien-3β-ol	41659

Note: Highlighted row is the metabolites those were mentioned in main text.

**Table 2:** Top 20 metabolites of *S*. *mekongi* with decreased level after PZQ treatment by multivariate analysis.

No.	Chemical formula	Exact mass	Ion adduct	Mass error (ppm)	Fold change			*p*-*value*	Potential metabolite	METLIN ID
					Low dose	Medium dose	High dose			
1	C_22_H_32_O_2_	329.2478	[M+H]+	1	-21.7	-49.6	-16.2	0.00047	Retinol Acetate	41508
2	C_22_H_35_NO_2_	346.2744	[M+H]+	1	-22.6	-33.9	-12.6	0.00047	Anandamide (20:5, n-3)	36743
3	C_19_H_28_N_2_O_4_	349.2115	[M+Na]+	2	-17.5	-37.3	-7.0	0.00047	Roxatidine acetate	85590
4	C_20_H_32_O_2_	305.2484	[M+H]+	3	-14	-29.2	-6.8	0.00047	8,11-eicosadiynoic acid	24087
5	C_20_H_35_NO_2_	322.2744	[M+K]+[M+Na]+	1	-10.2	-22.7	-4.9	0.00047	Anandamide (18:3, n-6)	36739
6	C_20_H_24_D_8_O_2_	335.2796	[M+H]+	0	-4.5	-9.1	-6	0.00047	Arachidonic Acid (D8)	3805
7	C_21_H_42_O_4_	381.2976	[M+Na]+	0	-3.0	-1.8	-2.6	0.00047	Hexadecyl Acetyl Glycerol	43452
8	C_20_H_38_O_3_	327.2897	[M+H-H_2_0]+	1	-2.5	-3.5	-2.4	0.00047	Lesquerolic acid	35494
9	C_29_H_50_O_4_	463.3770	[M+H]+	2	-2.8	-2.1	-1.5	0.00047	2α-(3-Hydroxypropyl)-1α,25-dihydroxy-19-norvitamin D3	42553
10	C_12_H_18_O_4_	227.1280	[M+H]+	1	-1.5	-4.9	-1.6	0.00047	Tuberonic acid	36075
11	C_18_H_30_O	263.2373	[M+H]+	1	-4.8	-11.0	-5.2	0.00054	9Z,12Z,15Z-Octadecatrienal	46529
12	C_22_H_38_O_5_	383.2793	[M+H]+	0	-2.3	-8.3	-5.4	0.00054	9-oxo-11R,15S-dihydroxy-16,16-dimethyl-13E-prostaenoic acid	36128
13	C_25_H_42_O_4_	407.3156	[M+H]+	0	-5.4	-2.9	-2.0	0.00054	Beta-monoacylglycerol (MG(0:0/22:4(7Z,10Z,13Z,16Z)/0:0))	62337
14	C_19_H_29_N_5_O_8_	456.2093	[M+K]+ [M+H]+ [M+H-HCOOH]+	1	-4.2	-5.0	-1.9	0.00054	Asp Pro Pro Gln	124533
15	C_25_H_49_NO_4_	428.3734	[M+H]+	0	-3.1	-3.4	-1.5	0.00054	DL-Stearoylcarnitine	5811
16	C_20_H_30_O	287.2364	[M+H]+	2	-3.5	-3.9	-2.9	0.00060	Retinol	215
17	C_31_H_46_O_7_	553.3133	[M+K]+	1	-1.5	-3.6	-2.6	0.00060	11-Ketorockogenin acetate	44214
18	C_22_H_36_O_2_	333.2794	[M+H]+	2	-33.7	-53.7	-14.0	0.00067	Arachidonic acid ethyl ester	404
19	C_22_H_33_N_9_O_6_	520.2641	[M+H]+	3	-5.1	-6.6	-3.2	0.00067	His His Ile Asn	154030
20	C_13_H_18_O_2_	207.1381	[M+H]+	1	-6.0	-6.3	-5.2	0.00074	Eremopetasinorone A	86413

Note: Highlighted row is the metabolites those were mentioned in main text

### Pathway analysis of the differential metabolites after PZQ exposure

To investigate deeper into the effects of PZQ on *S*. *mekongi*, the differential metabolites were subjected to pathway analysis according to KEGG pathway. The results (Table 3) showed that a number of metabolites belonging to Vitamin D3 biosynthesis, retinoate biosynthesis I, and resolvin D biosynthesis were significantly altered after exposure to low-, medium-, and high-dose PZQ, respectively (*p* < 0.05). A total of 11 pathways were affected by all three doses of PZQ, and these included anandamide degradation, aspirin-triggered lipoxin biosynthesis, bile acid biosynthesis (neutral pathway), C20 prostanoid biosynthesis, leukotriene biosynthesis, lipoxin biosynthesis, retinoate biosynthesis I, retinoate biosynthesis II, retinol biosynthesis, the visual cycle I, and zymosterol biosynthesis (Table 3). According to the pathway analysis, arachidonic acid metabolism strongly responded to PZQ exposure through three main pathways: anandamide degradation, leukotriene biosynthesis, and lipoxin biosynthesis. The significant alteration in anandamide degradation supports the finding of decreased anandamide level in PZQ-treated *S*. *mekongi*. In the anandamide degradation pathway, a molecule of anandamide is degraded into ethanolamine and arachidonic acid. Furthermore, leukotriene biosynthesis and lipoxin biosynthesis, which are processes within arachidonic acid metabolism, were also affected by PZQ exposure. Of the 75 metabolites in the arachidonic acid metabolic pathway, we identified 50 that were changed after PZQ exposure, reflecting the strong impact of PZQ on this pathway.

**Table 3:** Biological pathways altered by different concentrations of PZQ. Gray color represents alterations in biological pathways with *p* < 0.05.

Biological Pathway	PZQ concentration		
	Low	Medium	High
Anandamide degradation			
Aspirin-triggered lipoxin biosynthesis			
Bile acid biosynthesis, neutral pathway			
C20 prostanoid biosynthesis			
Leukotriene biosynthesis			
Lipoxin biosynthesis			
Retinoate biosynthesis I			
Retinoate biosynthesis II			
Retinol biosynthesis			
The visual cycle I			
Zymosterol biosynthesis			
Vitamin D3 biosynthesis			
Aspirin triggered resolvin E biosynthesis			
Aspirin triggered resolvin D biosynthesis			
Resolvin D biosynthesis			
Adenosine nucleotides degradation			
Adenosine ribonucleotides de novo biosynthesis			
Diphthamide biosynthesis			
Icosapentaenoate biosynthesis II (metazoa)			
tRNA splicing			
Ubiquinol-10 biosynthesis			
α-tocopherol degradation			
γ-linolenate biosynthesis			

Retinol biosynthesis, retinoate biosynthesis I and II, bile acid biosynthesis (S1 Fig) and C20 prostanoid biosynthesis were also impaired after all concentrations of PZQ treatment. We found that 15 out of the 25 metabolites involved in retinol metabolism showed changes in abundance following PZQ treatment, implying that PZQ had a substantial impact on this pathway. The pathways highlighted in this study might play important roles in the parasiticidal effects of PZQ against *S*. *mekongi* and might be used as target pathways for drug development.

### Alignment of target protein sequences

**Fig 8:**
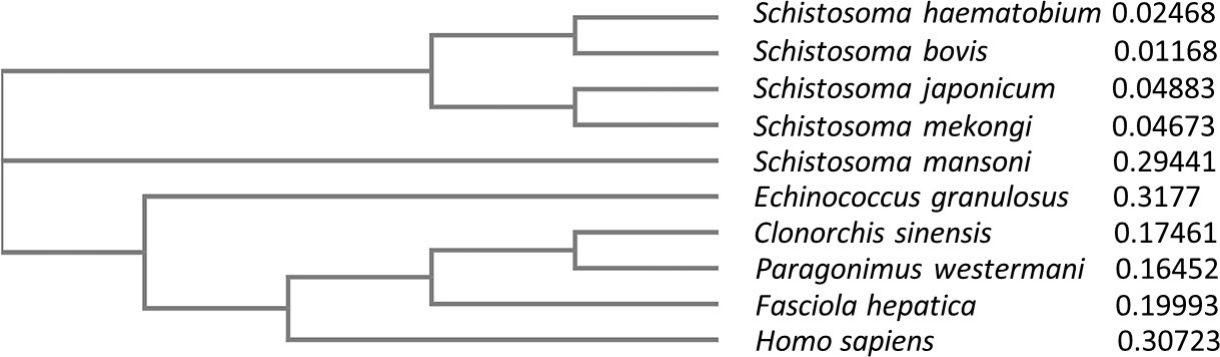
Phylogenetic tree of Fatty acid amide hydrolase from *S*. *mekongi*, other trematode species, cestode, and human. Five species of schistosome are grouped together and are distant from human.

According to the results of the pathway analysis, anandamide degradation and arachidonic acid metabolism are strongly involved in PZQ’s mode of action in schistosomes. Fatty acid amide hydrolase is an important enzyme in both mechanisms and plays an important role in the primary degradation of anandamide into ethanolamine and arachidonic acid. The fatty acid amide hydrolase sequences from *Homo sapiens* (NP_001432.2), *S*. *mekongi* (in house database), *S*. *japonicum* (TNN09110.1), *S*. *mansoni* (A0A3Q0KS85), *S*. *haematobium* (A0A095BTX5), *S*. *bovis* (RTG91480.1), *Fasciola hepatica* (A0A4E0S486), *Paragonimus westermani* (A0A5J4NW97), *Clonorchis sinensis* (H2KPN8), and *Echinococcus granulosus* (A0A068W6R9) were aligned to determine their phylogenetic tree and percentage identities (Fig 8 and S3 Table).

The alignment results showed that fatty acid amide hydrolase was conserved among *Schistosoma* species (Fig 8) (with 41.95%−90.44% identity), but the human and other parasite sequences were divergent (26.86–33.85% identity). Therefore, fatty acid amide hydrolase is a potential schistosomicide drug target.

## Discussion

Metabolomics is a powerful tool for identifying drug mechanism of action, which have been studied for many anthelminthic compounds targeting schistosomes, such as perhexiline maleate [19] and sclareol [31]. Moreover, this technique has been used to investigate drug resistance mechanisms and thus may lead to alternative treatments to combat the tolerance of pathogens. Paromomycin-resistant *Leishmania donovani* [32] and amphotericin B-resistant *L*. *mexicana* [33] are examples of species investigated for drug resistance mechanisms using metabolomics. Presently, there is little information on PZQ’s mode of action in schistosomes, meaning it is difficult to overcome PZQ resistance. Therefore, through this study, we aimed to apply metabolomics to understand PZQ mechanism of action in *S*. *mekongi*. According to our metabolomic data, PCA analysis revealed deviation of all treatment groups from control group. There were 3,849, 1,848, and 3,778 metabolites of *S*. *mekongi* were different from baseline at low, medium, and high concentrations of PZQ, respectively. The number of altered metabolites of *S*. *mekongi* corresponded to those in a previous report of *S*. *mansoni* metabolomic profiling after PZQ treatment, which described 2,756 metabolite changes and the additional effects of PZQ on the glycolysis, tricarboxylic acid cycle, and pentose phosphate pathways [31]. One interesting point regarding number of altered metabolites is lower number in medium-dose group. Alteration of metabolites did not linearly respond to doses of the treatment, as observed from several studies such as Fernandes, *et al*. [34] and Zhao, *et al*. [35]. In the PCA analysis, the 2 replications of medium-dose group were separated from others, we hypothesized that the deviation may come from biological differences between replications because all experiments were performed using exactly same protocols and conditions. Although, there were outliers in the medium-dose group, the centroids of PCA score plot of each PZQ dose clusters were significantly different. The centroid of low-dose, medium-dose and high-dose on PCA score plot were (3011.11, -2643.879), (-6516.275, -3540.422), and (4011.466, -3896.345), respectively.

Level of PS (14:0/12:0) or 1-tetradecanoyl-2-dodecanoyl-glycero-3-phosphoserine, increased in all three PZQ treatment conditions, is a major constituent of the schistosome tegument [10] and is involved in cell-cycle signaling, specifically, during apoptosis [36]. PS was reported to fluctuate after PZQ treatment and during the development of *S*. *mansoni* stages [37]. The excretory/secretory products of schistosomules and adults were found to contain PS as a host immunomodulator [38]. PS is a substrate of *Schistosoma* ABC multidrug transporter, which is hypothesized to be a protein associated with PZQ resistance [39]. The translocation of PS via ABC transporters from the inner to the outer side of the cell membrane is the hallmark of cellular apoptosis and is believed to be a signal for phagocytes [40]. Furthermore, the increase in PS after PZQ treatment might result in the exposure of the parasite to host effector immune cells, antibodies, and toxic molecules and radicals [41]. Phosphatidylserinedecarboxylase (PSD) is an enzyme that catalyzes PS into phosphatidylethanolamine, and using a PSD inhibitor can eliminate the malarial parasite [42] and promastigote stage of *L*. *infantum* [43]. Because of the various biological roles of PS and PSD in several parasites, they have been proposed as potential schistosome drug targets [10], [44]. PS is also a metabolite of arachidonic acid metabolism that, in our study, was demonstrated to be a significant differential pathway, and arachidonic acid was metabolite of *S*. *mekongi* that altered after PZQ exposure. In general, arachidonic acid plays critical roles in signaling [45], inflammatory responses, and the immune system [46]. In mammalian hosts, arachidonic acid is mainly synthesized from phospholipids. However, parasitic helminths cannot *de-novo* synthesize their own long-chain polyunsaturated fatty acids from acetate. Because this type of fatty acid is a component of phospholipids, parasites need to obtain them from the host to produce arachidonic acid [47,48]. Arachidonic acid is a starting material in the synthesis of two kinds of essential substances—the prostaglandins and leukotrienes—both of which are also unsaturated carboxylic acids. High amounts of arachidonic acid might be required for the production of prostaglandins and leukotrienes, which induce stress and trauma in *S*. *mekongi*. [49]. Supplementation with arachidonic acid has been suggested as a novel method for the treatment of schistosomiasis [50]. *In vitro* and *in vivo* exposure to arachidonic acid killed *Schistosoma* spp. effectively via the mechanisms of spine destruction, membrane blebbing, and disorganization of the apical membrane structure [51]. A reduction in worm burden and egg load was observed after the administration of arachidonic acid to hamsters [52]. Arachidonic acid supplementation has been tested in Egypt using school-aged children, and the findings showed that the treatment efficacy of this compound was not different from PZQ in lightly infected children. Interestingly, a combination of arachidonic acid and PZQ enhanced parasiticidal activity to 100% [53,54]. Thus, arachidonic acid and arachidonic acid metabolism are promising targets for the development of drugs against schistosomes.

Generally, PZQ causes severe spasms and paralysis of *Schistosoma* muscles, and this paralysis is caused by a rapid Ca^2+^ influx inside the schistosome. Therefore, schistosome calcium ion channels are currently proposed targets of PZQ [55]. On the basis of our metabolomic data, anandamide was the most decreased metabolite after exposure to all PZQ concentrations. Anandamide is a secondary messenger that binds to type-1 cannabinoid receptors and has been shown to directly modulate various ion channels, including calcium ion channels [30]. Anandamide suppresses calcium overload through the inhibition of the Na^+^/Ca^2+^ exchanger [56]. The downregulation of anandamide after PZQ treatment could reduce the inhibition of calcium ion channels, leading to Ca^2+^ influx into schistosomes, affecting their muscles. When school children in Ethiopia were treated for *S*. *mansoni*, most (80.7%) reported three or more side effects, such as headache, dizziness, nausea, tiredness, weakness, loss of appetite, and vomiting [57]. In humans, anandamide is dominantly produced in the brain, and it shows neuromodulatory effects and influences vital brain functions [58]. The modulation of anandamide in the human brain produces changes in appetite, dizziness, and lightheadedness [59]. As such, PZQ might also mediate anandamide levels in the human brain and cause undesired side effects to the patients. Regarding invertebrates, PZQ has a potent effect on trematodes but less of an effect on nematodes. Although anandamide has been detected in both nematodes and platyhelminths [60], there are some differences in terms of the protein structure of fatty acid amide hydrolase, a key enzyme for anandamide degradation in both worms. In the phylum Nematoda, this enzyme contains Phe and Trp in the active region; whereas, in Platyhelminthes, the enzyme is predicted to have Tyr and Cys substitutions in the active region [61]. Therefore, the different active sites of fatty acid amide hydrolase may be responsible for the incompatible activity and stoichiometry in regulating the anandamide degradation pathway. Our multiple alignment analysis supported this hypothesis by showing that amino acid sequence of this enzyme is conserve among *Schistosoma spp*. (Fig 8 and S3 Table). Hence, the dissimilarities in anandamide degradation may correspond to the different impact the PZQ has towards trematodes and nematodes. After analyzing our findings, we hypothesized that fatty acid amide hydrolase regulates anandamide levels and plays important roles in *Schistosoma* ion channel and signal transduction regulation. This protein could be a potential target for schistosomiasis treatment.

A number of metabolites in retinol metabolism were decreased after PZQ treatment. Retinol, also known as vitamin A1-alcohol, is important for growth, development, the immune system, and vision [62]. In *S*. *japonicum*, retinol metabolism is associated with meiosis processes and the growth of worms [63]; therefore, the downregulation of retinol metabolism by PZQ treatment may inhibit egg production and reduce the growth of *S*. *mekongi*.

Although our study successfully highlighted drug targets, there are some limitations to be aware. We performed metabolomic analysis only for paired worms, without data of unpaired male and female parasites. The altered metabolome was a result of the interaction of the two sexes, as such a more detailed investigation of the individual sexes will be required in future studies. To convert LC-MS raw data into metabolite identification and abundances, peak retention time, mass and MS/MS fragmentation pattern provide great specificity to match peaks with metabolites. However, the presented metabolites were only "hits" or "features" reported by XCMS software. A unique metabolite does not always correspond to a feature. [64]. This type of artifact is commonly observed when the peak resolution is not clear. According to this limitation, the actual number of identified metabolites may be lower than reported in this study. In addition, the labeled standards to ensure the chemical structure of the metabolites "identified" by comparing MS/MS spectra with METLIN databases were not performed. Therefore, all metabolites reported in this study were referred to the "putative identification”.

In summary, we treated *S*. *mekongi* with increasing doses of PZQ and performed metabolomic analysis to identify the mechanism of action and important biological pathways associated with the effect of PZQ. These pathways could be potential candidates for anthelminthic drug development. Pathway analysis of differential metabolites revealed that arachidonic acid metabolism was the most prominent pathway involved in the schistosomicidal effects of PZQ. Novel drugs targeting PS (14:0/12:0), anandamide, and arachidonic acid metabolism may be effective approaches to overcome the problem of PZQ-resistance and schistosomiasis in the future.

## Supporting information

S1 Fig *S*. *mekongi* worms after PZQ treatment. (A). Control. (B) Low dose PZQ treatment. (C) Medium dose PZQ treatment. (D) High dose PZQ treatment. The worms in treatment groups were bended and coiled comparing to the control group.
(TIF)

S1 Data Metabolomic raw data. The.wiff files of each sample were generated by LC-MS. (A) Control replication 1. (B) Control replication 2. (C) Control replication 3. (D) Low dose PZQ treatment replication 1. (E) Low dose PZQ treatment replication 2. (F) Low dose PZQ treatment replication 3. (G) Medium dose PZQ treatment replication 1. (H) Medium dose PZQ treatment replication 2. (I) Medium dose PZQ treatment replication 3. (J) High dose PZQ treatment replication 1. (K) High dose PZQ treatment replication 2. (L) High dose PZQ treatment replication 3.
(ZIP)

S2 Data Detailed metabolic extraction protocol. The workflow of metabolite extraction from *S*. *mekongi* worms was presented step-by-step.
(DOCX)

S3 Data Fatty acid amide hydrolase protein sequences. The amino acid sequences of *Homo sapiens*, *Schistosoma mekongi*, *Schistosoma japonicum*, *Schistosoma mansoni*, *Schistosoma haematobium*, *Schistosoma bovis*, *Fasciola hepatica*, *Paragonimus westermani*, *Clonorchis sinensis*, and *Echinococcus granulosus* fatty acid amide hydrolase that were used for alignment were provided.
(DOCX)

S4 Data Fatty acid amide hydrolase protein sequence alignment file. The.clustal_num file result generated from Clustal alignment analysis was provided.
(CLUSTAL_NUM)

S5 Data Protein sequence alignment result from Clustal Omega software. The.txt file result generated from Clustal alignment analysis was provided.
(TXT)

S1 Table Top-10 metabolites of *S*. *mekongi* with increased level after low-, medium-, and high-dose PZQ treatment. The pairwise comparison was performed on these results. Phosphoserines were highly produced in *S*. *mekongi* after low-, medium-, and high-dose PZQ treatment.
(DOCX)

S2 Table Top-10 metabolites of *S*. *mekongi* with decreased level after low-, medium-, and high-dose PZQ treatment using pairwise comparisons. The pairwise comparison was performed on these results. Anandamide was highly produced in *S*. *mekongi* after low-, medium-, and high-dose PZQ treatment.
(DOCX)

S3 Table Alignment of *Homo sapiens*, *S*. *mekongi*, *S*. *japonicum*, *S*. *mansoni*, *S*. *haematobium*, *S*. *bovis*, *Fasciola hepatica*, *Paragonim us westermani*, *Clonorchis sinensis*, and *Echinococcus granulosus* fatty acid amide hydrolase sequences. Numbers in the table represent percentage identity matrices. Fatty acid amide hydrolases among parasites had high percent similarities.
(DOCX)
