# Supplementary figures and images for: Metabolomics reveal alterations in arachidonic acid metabolism in Schistosoma mekongi after exposure to praziquantel
Source: PLoS Negl Trop Dis. 2021 Sep 2;15(9):e0009706. doi: 10.1371/journal.pntd.0009706 (PMC8412319; doi:10.1371/journal.pntd.0009706)

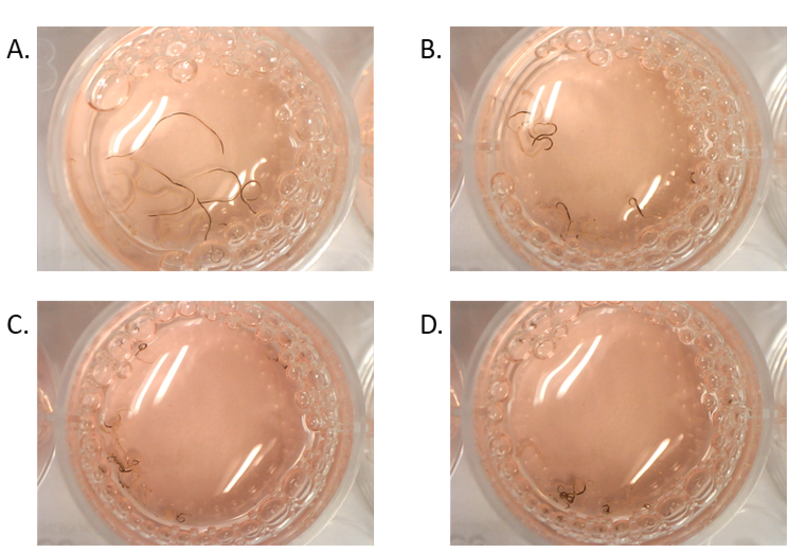

Supplement: S1 Fig — (A). Control. (B) Low dose PZQ treatment. (C) Medium dose PZQ treatment. (D) High dose PZQ treatment. The worms in treatment groups were bended and coiled comparing to the control group. (TIF) [file pntd.0009706.s001.tif]
