# Supplementary material for: Metabolomics reveal alterations in arachidonic acid metabolism in Schistosoma mekongi after exposure to praziquantel
Source: PLoS Negl Trop Dis. 2021 Sep 2;15(9):e0009706. doi: 10.1371/journal.pntd.0009706 (PMC8412319; doi:10.1371/journal.pntd.0009706)
Supplement: S2 Data — The workflow of metabolite extraction from S. mekongi worms was presented step-by-step. (DOCX) [file pntd.0009706.s003.docx]

**Protocol for metabolite extraction**

• After washing, all worms from each condition are transferred into 1.5-mL microcentrifuge tubes

• Add 500 μl methanol and homogenize

• Snap-freeze in liquid nitrogen and thaw

• Centrifuge at 800 × g for 1 min at 4 °C

• Collect supernatant and place in a new tube

• Extract pellet again with the same protocol

• Pool supernatant from the second extraction to the tube containing the supernatant from the first extraction

• Resuspend pellet with 250 μl of deionized H2O

• Snap-freeze in liquid nitrogen and thaw

• Centrifuge at 15,000 × g for 1 min at 4 °C

• Collect supernatant and pool with the previous tube

• Centrifuge pooled supernatants at 15,000 × g for 1 min at 4 °C

• Transfer supernatant to a new tube

• Dry supernatant in a speed vacuum
