# Supplementary material for: Metabolomics reveal alterations in arachidonic acid metabolism in Schistosoma mekongi after exposure to praziquantel
Source: PLoS Negl Trop Dis. 2021 Sep 2;15(9):e0009706. doi: 10.1371/journal.pntd.0009706 (PMC8412319; doi:10.1371/journal.pntd.0009706)
Supplement: S3 Data — The amino acid sequences of Homo sapiens, Schistosoma mekongi, Schistosoma japonicum, Schistosoma mansoni, Schistosoma haematobium, Schistosoma bovis, Fasciola hepatica, Paragonimus westermani, Clonorchis sinensis, and Echinococcus granulosus fatty acid amide hydrolase that were used for alignment were provided. (DOCX) [file pntd.0009706.s004.docx]

**Amino acid sequence of fatty acid amide hydrolase**

| **Species** | **Sequence (FASTA)** |
| --- | --- |
| Human  (*Homo sapiens*) | >NP_001432.2 fatty-acid amide hydrolase 1 [Homo sapiens]  MVQYELWAALPGASGVALACCFVAAAVALRWSGRRTARGAVVRARQRQRAGLENMDRAAQRFRLQNPDLD  SEALLALPLPQLVQKLHSRELAPEAVLFTYVGKAWEVNKGTNCVTSYLADCETQLSQAPRQGLLYGVPVS  LKECFTYKGQDSTLGLSLNEGVPAECDSVVVHVLKLQGAVPFVHTNVPQSMFSYDCSNPLFGQTVNPWKS  SKSPGGSSGGEGALIGSGGSPLGLGTDIGGSIRFPSSFCGICGLKPTGNRLSKSGLKGCVYGQEAVRLSV  GPMARDVESLALCLRALLCEDMFRLDPTVPPLPFREEVYTSSQPLRVGYYETDNYTMPSPAMRRAVLETK  QSLEAAGHTLVPFLPSNIPHALETLSTGGLFSDGGHTFLQNFKGDFVDPCLGDLVSILKLPQWLKGLLAF  LVKPLLPRLSAFLSNMKSRSAGKLWELQHEIEVYRKTVIAQWRALDLDVVLTPMLAPALDLNAPGRATGA  VSYTMLYNCLDFPAGVVPVTTVTAEDEAQMEHYRGYFGDIWDKMLQKGMKKSVGLPVAVQCVALPWQEEL  CLRFMREVERLMTPEKQSS |
| Common liver fluke  (*Fasciola hepatica*) | >tr\|A0A4E0S486\|A0A4E0S486_FASHE Fatty-acid amide hydrolase 1 OS=Fasciola hepatica OX=6192 GN=D915_000201 PE=3 SV=1  MVYKIPTPKFHKGLWVAGGLGLAYFVYQAVSSMLIRREWAKRRDRKRKENAAAKAQFAEY  LRQNPMKSTDVEAIVSKPFAEILSSLKSGEITPEKALLAYQHKAFAVDQRCNCVVEFLYP  DLKLVNLKGPFAGMPVSVKDNYKIKGHESCAGMAYFLTGPAKEDSVIISVLYSLGAVPFV  RTNVPQSMLSVLSWNPINGVTLNPLALDRSPGGSSSGEAALVGGGGSILGFGTDLGGSIR  LPSAMCNIVGFKPTANRISQRKMLSFKRLMTTFSCCGPLARDVETCITAMQCIWNSQLHH  DLDYMTPPVPYTFTPPDRPLRIGYFTYDGSFKAVPAAERAVLHIKSRLEAMGHKLIPWQP  PYPGSECFEMFLTALFVDGGARLVEMLQYDEVEKSMFLGLKVFKSWRLTRRIKWILAKLF  THPDNLIYMRAVNYVTDVPTLTQHINALEEFRYRWYDNWRESNLDAVLCPAFGMAAPVPV  RTTRSFAGMLTYLHLFNITNMPAGSVPSGICVTEADLAPLRASLGQKDDNKPVKLAPGVT  PYPIDRPWQKAAAELQVDTLGMPVGVQVAAAPWHDEMCLHVMREVERAARGVSQPGPTN |
| Chinese liver fluke  (*Clonorchis sinensis*) | >tr\|H2KPN8\|H2KPN8_CLOSI Fatty-acid amide hydrolase 1 OS=Clonorchis sinensis OX=79923 GN=CLF_102336 PE=4 SV=1  MWFSGNHRQLLKGLLYIGGACVAYYWVSSLFHGFFMRREYARRRERKRQEKAAAKRAFGN  YLNKHPMPEEDIAKITSKPFLELVHMLKSGQLTPERVLLAYQHKAFEVDARCNCVIEFLY  PDFSEINLNGPFSGVPISLKDNYSVKGCESYAGMACHLTGPADKDSVMVTVLKSLGAVPF  VRTGVPQAMLSVLSSNPIDGTVLNPLDLSRSPAGSSSGEGALVGGGGSILGFGTDLGGSI  RLPAAMCNCVGFKPTPSRLSKRLMLNTNRFMTTDSSCGPIAREVDTCITLMKALFDSPLH  HELDYFGAPLCYSTSISTDRRLRIGYFVDDGSTPPVPAAQRAVLVVKDKLAALGHELVPW  QPPVTGTEWLKMFLTTLVTDGGMPLLQALRYDVVQPPLNQSLKIYKSWWITRYFQQFIAR  LLGRHTELQLMQACCAVHDIPSLMQHLEDLKAFRQRIYDHWNQERIDAVICPAFGMAAPI  LAKSNRKFTEMLTYLNLFNVTNMPAGCLPSGISVDENDIEALKNSAPPNGTGSPWSKDVI  ELQADTLGFRVSVQVAAAPWRDELCLHVMKEVEKAVRL |
| Lung fluke  (*Paragonimus westermani*) | >tr\|A0A5J4NW97\|A0A5J4NW97_9TREM Fatty acid amide hydrolase (Fragment) OS=Paragonimus westermani OX=34504 GN=DEA37_0009441 PE=4 SV=1  IMYVPQHHRLAFKGLMVLGGVGILSYWLSTGIYSTIMRRRYAARRERKRTEKAQAKTKFA  DYLRRNPMSPEKITSITSKPYMALVQALKQGEINPEEALLAYQHKAFEVDKRCNCVVEFL  YPNMSEINLSGPFAGAPISLKENFSVKGCESYAGMAYFLTGPAQDDAVLVTVLKALGAVP  FVRTTVPQAMFSVLSSNPIDGTTLNPLDPTRSPAGSSSGEAALIGGGGSGLGFGTDLGGS  IRLPAAMCNIVGFKPTPGRLSQRLMLSINRFMTTTSSCGPLARDVDTCIAAMKTLCDSPL  LHSLDYFNPPLPYSTELPLNRPLRIGFFTYDGSFTPVPAAQRAVLHVKEKLEALGHKLIP  WAPPYLGHEWLTIFLTALMVDGGSSLLNVLKYDVVEPTLATGLRFYKSSWLLRYLKLLYV  KLLGNPEDLHFLRACCTVYDIPSLMEHTVHLKRFRQCIYDHWNEAQLDAVICPAFGMAAP  IPVTTSRKFTGMLSYQNLFNITNMPAGCLPSGIRVTEQDLTEMRALLNKRSVWHQAAHDL  QLDTLGFSVSVQVAAAPWRDELCLHVMREVERAVQS |
| *Schistosoma mansoni* | >tr\|A0A3Q0KS85\|A0A3Q0KS85_SCHMA Putative fatty-acid amide hydrolase OS=Schistosoma mansoni OX=6183 PE=3 SV=1  MEQSLRFCWYLLPTLLASITKYAVRLILLPIFIIFILGHAVKYFKKKSKLRIKLLRKRQS  ITQRMDQLKEHLSSTKSTSNIDLLSVTDLNLDTIQERLVEGKFTSVDLLHAYQMKALQLY  DSGNSGICEFLDEAEELAVDLANMNRLPTSKQTLVGIPVSLKELCSIKGYDITFGLINRC  NKPSHKDCCIVEVLRHEGAIPFVLTATSQTALSLSGINPVFGDMSNPHSSEHETGGSSSG  EGVLLGLRGSPVGIGTDLAGSIRIPSVFCGLVGLKPTTNRISSKGVGVIGHKKSILLRVC  VGPMGRRVDDLAKLMRTLLTTKMFQMDPYVPPLLFNDMIYAGIDKPKLTIGYYVTLEDPL  IITSVPSVRRIVNESVDILRQRGHILLPFHPPNIKWAYELGMKAISVDTKYHVQEALFAE  PLNEHTKFLRLLINTPHWLKVMIDKLLTIIFGRPAAVTSFLDGPRGEAETLNLISDIEFY  RHEFQRAMEEAGNLDAIICPVFPFPAFPKSAKSMFVTPAVAYTVLFNMLDYPAGTVPMGY  VNKEDVQNSNVLAEEYKKVGNRYLYNVFKYQETSEGLPVGVQIIGKPWQEERVLYVMKEL  ETSRTLTE |
| *Schistosoma haematobium* | >tr\|A0A095BTX5\|A0A095BTX5_SCHHA Fatty acid amide hydrolase 1 OS=Schistosoma haematobium OX=6185 GN=MS3_00394 PE=3 SV=1  MDSQSKQLSLNNNNELTSMSLSQLREKLHNRSITSVDLLDAYQIRGLELLRTRSNCISEI  IYEADVYAILADSSRDSEGGHVSSIHGIPIALEEIFPIHGYDHTMGYTIRTNRGAEDDCI  LVKALRDCGAIPVILTNVKQKILGLSANNPITGLTSHPTHPGRACVSGMGPLLVHKGSPL  AVGFDILGEARLSAAFCGKAALKPTPYRMSNKDLKLPIELPEDLLPVPSPMGHRIEDIVD  VLKSLWTSNMFAHDCTLCPMAFNDKEYKSIAEGNRKLKIGFYSNFDGLVKASPSVQRVMS  EIRDELSRQGHEVVDFALPTPSKAYQLTISLLASYMEPESLKLLYIHGNGDILVDYRQRL  LHLFYALPRFLRHRIAEWRAEGIKKDYPATAVVLRGLGYSQSRKTLINQINDYKQEFFSL  WNEVELDVLVCPTAPIPAPWDDSPSYVTNCVLPFTCLYNLLGCPAGTLSVGRVEKCDLQA  CGDISDNNFKSSQLDIMFSEQHKRSEGLPIGVQVVAKPWNDDQTIYIIVNIHLQSAVQIT  LNRSKLTSVIKSPKQLCPDDIRKMEKYNVERVYVGAFFYRCLNTWPTSSKGQECSVIELF  NEKANFSIIVSPIGADEPVIGVAKSRLTKEMANSGVDSVLIVSKEYFISLMRHPVRHLLV  AWSLGLISWKIITCIRTYQNQKLLKSKQIRIADNTEKMRKLLSKLTHSSIPMAICSENLS  YLCEQIKKKRVTPVDVLHAFQFRALQLQDNNNSGIAEFILEAEEYAANLTKSPMNIDEQS  GLYGIPISIKEGIAIRGYDVTMGIIKRCSQPIDEDCVLIKVLRSVGSIPFVTTVTTQLCR  TLDSFHSIYNDAKNPFNKSRLPGGSSSGEAVLLAQHGSPVGIGGDIAGSIRIPCAFCNLA  GLKPTSGRLSLLGFVSAAKKSVLYLSPCLGPMARKVDDLACVMRALLCPTMFDLDPYVIP  MSFDQVSYEGKNRKQLVIGYYLNFDDPNLIQVLDVNQQVVEKAAKALESAGHRLVKFTVP  DPYKAFILGLHALFADGGQELRSHLRGEPLSPHIKLISTVTRIPDILKPIVGFISKHFIG  KPVCPSGAFQKLGSGQAAVNLISEIKAYRYEFAKAWNNAGPLDALICPVFPYPAPPEDAS  QVYISPSIIYTFLYNILDYPAGAVPAGFVTEDDVRNSLMKSESLYSTGDSYMSKVYGLLN  GGENLPLSIQVVGKPYHEETVLRVMREIESSFSQQTES |
| *Schistosoma japonicum* | >TNN09110.1 Fatty acid amide hydrolase 1 [Schistosoma japonicum]  MYNTVNTTVGVILKISEWCASFLTKPSTRRIILVLSFGLVSWKIVASIRIHQNQKLLKSKQRRITNNVEK  LRKKLSNFSQSYTPCDVYGKSLSFICDQVKTGKMTPIDILHSFQLKALQLQDDGNSGIAEFILEAEDYAV  NLMKPSVDINKESGLYGIPISIKEGISICGYDATMGIIKRCNQPMNEDCILVKVLKHVGSVPFVTTVTTQ  LCRTLDSFNCVYHGAKNPFDKSRMPGGSSSGEAVLLAQCGSPVGIGTDIAGSIRIPCAFCGLAGLKPTLR  RLSTSGLASTATKSVLYLSPCLGPMGRKVDDLACVLRALLCPVMFDLDPYVVPLHFDQMSYEGKNKRQLV  IGYFTSFDDPNLIETLDVNQRIVEEAAKALESAGHKLVKYTVPHPYEAFILGLRALFADGGQELQSHLQN  EPLSPQLKFLKTIIGLPDFLKPVIGSVSSYFVGKPISISSALLKLKSGQAAINLLAEIDAYRYEFARVWD  EAGPLDVLICPVSPYPAPPEDTLPLFISPSIIYTFLYNIVDYPAGAVPAGFVKKEDVHNSLMKSELLHSS  GDTYLSKVYKLLDGGENLPLSIQVVGKPYHEETVLRVMREIENCFLKTES |
| *Schistosoma mekongi* | >in-house database  MVNTTVGAILNLSEWCANFLTKPSTRHIILVLSFGLVSWKIVASIRIHQNQKLLKSKQKRITN  NVEKIRKNLSNFSQSHTLCDVYSKSLSFICEQINTGKMTPIDILHSFQLRALQLQDDGNSGIA  EFILEAEDYAINLMKSSVDINKESGLYGIPISLKEGISVCGYDATMGIIKRCNQPVDKDCVLVK  VLKSVGSVPFVTTMTTQLCRTLDSFNCVYHDAKNPYNKSRMPGGSSSGEAVLLAQCGSPL  GFGTDIAGSIRIPCAFCGLAGLKPTFRRLSTLGLASTATKSVLYISPCIGPMAKKVDDLACVLR  ALLCPAMFDLDPYVVPMHFDQMSYEGKNKRQLVIGYFTNFDDPDLIQTLDVNQRIVEEAA  KALENAGHKLVKYTVPHPYEAFILGLRALFADGGQELQSHLQNEPLSPQLKFLKTIIGLPDFL  KPVIGFVSRYCIGKPVGISSALLKLRSGQAAVNLLAEIDAYRYEFARAWDEAGPLDVLICPVS  PYPAPPDDAMPLFISPSIIYTFLYNILDYPAGAVPAGFVKKEDVHNSLMKSELLHSFGDAYLS  KVYKLLDGGENLPLSIQVVGKPYHEETVLRVMREIENCFLKTES |
| *Schistosoma bovis* | >RTG91480.1 fatty acid amide hydrolase [Schistosoma bovis]  MRIADNTEKMRKILSKLTHSSIPMAICSENLSYLCEQIKKKRMTPVDVLHAFQFRALQLQDNNNSGIAEF  ILEAEEYAANLTQSPMNIDEQSELYGIPISIKEGIAIRGYDVTMGIIKRCNQPIDEDCVLIKVLRSVGSI  PFVTTVTTQLCRTLDSFHSIYNDAKNPFNKSRLPGGSSSGEAVLLAQHGSPVGIGGDIAGSIRIPCAFCN  LAGLKPTSGRLSLLGFVSAAKKSVLYLSPCLGPMARKVDDLACVMRALLCPTMFDLDPYVIPMSFDGVSY  EGKNRNQLVIGYYLNFDDPNLIQVLDVNQQVVEKAAKALESAGHRLVKFTVPDPYKAFILGLHALFADGG  QELRSHLRGEPLSPHIKLISTVTRIPDILKPIVGLISKHFIGKPVCPSGAFQKLGSGQAAVNLISEIEAY  RYEFAKAWNNAGPLDALICPVFPYPAPPEDASQVYISPSVIYTFLYNILDYPAGAVPAGFVTEDDVRNSL  MKSESLYSTGDSYMSKVYGLLNGGENLPLSIQVVGKPYHEETVLRVMREIETWNNAGPLDALICPVFPYP  APPEDASQVYISPSVIYTFLYNILDYPAGAVPAGFVTEDDVRNSLMKSESLYSTGDSYMSKVYGLLNGGE  NLPLSIQVVGKPYHEETVLRVMREIESSFSQQTES |
| Hydatid worm  (*Echinococcus granulosus*) | >tr\|A0A068W6R9\|A0A068W6R9_ECHGR Fatty acid amide hydrolase 1 OS=Echinococcus granulosus OX=6210 GN=EGR_03165 PE=3 SV=1  MNLTDSTLEDFVSEFIAAWKRVQLWEIFGSIVLIYFTFRLLRRILLRRKVRRLLAEKQEL  LQEARARLLDRVQGDPPASTLKDLSALEIHDRLQFQEMTPFEALRIYQKRVVDVLGSNCV  CDVIEEAEATAKSIDSNFHSPIRGLPVSVKENIAIAGYDSSMGLVSRLLKPVRRDGVLIQ  VIRGVGAVPFVTTTMSPTGYCLDGSTEIFGKQRNPYDAKRLTGGSSSGEAVLIAKGASPL  GFGTDLAGSVRLPAAFCGICALKPTTHRVSTMGVESVSETGCIGLRPVFGPMARHTRLLT  DAMRAILTSVMFNLDPRTPHLPFHEGVFSSSKPLLIGFYTNFGGDIAMKVVPSVESALFR  AKAGLQAKGHTLVDFEVPEPNKVVCLTVKSMLGDGGVAFCEAARCEPANPRLRRTRFLLG  VPALLRRMVGWFVSTFFSKSIGAVFHASTGCQTALEVFTVSKEVETYREEFAKAWAAAKI  DVLLGPAFPFPAPLEGTPDVLLAGAVTFTSIYNLLDYPAGVVPVSKVNESDVEACRKLEG  EYRKAGDWLNATHAKLQSDTKGLPVAVQVVGKPFDEETVLRVMKEIEESLPLE |
