# Supplementary material for: Metabolomics reveal alterations in arachidonic acid metabolism in Schistosoma mekongi after exposure to praziquantel
Source: PLoS Negl Trop Dis. 2021 Sep 2;15(9):e0009706. doi: 10.1371/journal.pntd.0009706 (PMC8412319; doi:10.1371/journal.pntd.0009706)
Supplement: S1 Table — The pairwise comparison was performed on these results. Phosphoserines were highly produced in S. mekongi after low-, medium-, and high-dose PZQ treatment. (DOCX) [file pntd.0009706.s007.docx]

**S1 Table. Top-10 metabolites of *S. mekongi* with increased level after low-, medium-, and high-dose PZQ treatment using pairwise comparisons.**

| **Number** | **Chemical formula** | **Exact mass** | **Ion adduct** | **Mass error (ppm)** | **Fold change** | ***p*-*value*** | **Potential metabolite** | **METLIN ID** |
| --- | --- | --- | --- | --- | --- | --- | --- | --- |
| Low dose PZQ treatment | | | | | | | | |
| 1 | C_13_H_23_NO_2_ | 225.17 | [M+H]+ | 1 | 51.7 | 0.00025 | (8S,Z)-6-((S)-3-hydroxy-2-methylpropylidene)-8-methyloctahydroindolizin-8-ol | 263684 |
| 2 | C_35_H_68_NO_10_P | 693.45 | [M+H]+ | 2 | 50.1 | 1.1329e^-6^ | 1-dodecanoyl-2-heptadecanoyl-glycero-3-phosphoserine (PS (12:0/17:0)) | 77714 |
| 3 | C_11_H_22_N_2_O_3_ | 230.16 | [M+H]+ | 0 | 44.2 | 4.3446e^-7^ | Isoleucyl-Valine | 85825 |
| 4 | C_34_H_66_NO_10_P | 679.44 | [M+H]+ | 2 | 38.5 | 1.2567e^-6^ | 1,2-ditetradecanoyl-sn-glycero-3-phosphoserine (PS (14:0/14:0)[U] | 40794 |
| 5 | C_35_H_66_NO_10_P | 691.44 | [M+H]+ | 2 | 36.7 | 9.3181e^-7^ | 1-dodecanoyl-2-(9Z-heptadecenoyl)-glycero-3-phosphoserine (PS (12:0/17:1(9Z))) | 77715 |
| 6 | C_33_H_64_NO_10_P | 665.42 | [M+H]+ | 2 | 34.6 | 0.00055 | 1-O-hexadecanoyl-2-O-(9-carboxyoctanoyl)-sn-glyceryl-3-phosphocholine (PAz-PC) | 63020 |
| 7 | C_35_H_66_NO_10_P | 691.44 | [M+H]+ | 2 | 31.0 | 7.3599e^-8^ | 1-tridecanoyl-2-(9Z-hexadecenoyl)-glycero-3-phosphoserine (PS (13:0/16:1(9Z))) | 77737 |
| 8 | C_29_H_56_NO_10_P | 609.36 | [M+H]+ | 2 | 28.8 | 1.4098e^-6^ | 1-palmitoyl-2-glutaryl phosphatidylcholine (PGPC) | 45374 |
| 9 | C_35_H_68_NO_10_P | 693.45 | [M+H]+ | 2 | 28.2 | 8.7550e^-8^ | 1-tridecanoyl-2-hexadecanoyl-glycero-3-phosphoserine (PS (13:0/16:0)) | 77736 |
| 10 | C_33_H_64_NO_10_P | 665.42 | [M+H]+ | 1 | 28.1 | 5.5218e^-8^ | 1-dodecanoyl-2-pentadecanoyl-glycero-3-phosphoserine | 77711 |
| Medium dose PZQ treatment | | | | | | | | |
| 1 | C_27_H_44_O_2_ | 400.33 | [M+H]+ | 0 | 203.9 | 0.17646 | Cholesta-5,7-diene-1,3-diol | 41685 |
| 2 | C_34_H_66_NO_12_P | 711.43 | [M+H]+ | 3 | 14.5 | 0.00365 | Lipid X | 3916 |
| 3 | C_35_H_61_NO_12_ | 687.41 | [M+Na]+ | 4 | 14.5 | 0.00380 | Oleandomycin | 44103 |
| 4 | C_32_H_62_NO_10_P | 651.41 | [M+H-CH_2_]+ | 2 | 12.6 | 0.00257 | 1-tetradecanoyl-2-dodecanoyl-glycero-3-phosphoserine (PS (14:0/12:0)) | 78595 |
| 5 | C_20_H_34_O_3_ | 322.25 | [M+H]+ | 1 | 10.5 | 5.0623e^-6^ | 1,6,10,14-Phytatetraene-3,5,9-triol | 985105 |
| 6 | C_45_H_62_O_4_ | 666.46 | [M+Na]+ | 5 | 10.4 | 0.010 | Trans-Geranylgeranylbixin | 89894 |
| 7 | C_41_H_64_O_12_ | 748.43 | [M+Na]+ | 1 | 10.2 | 0.00743 | Flaccidin B | 67291 |
| 8 | C_30_H_58_NO_10_P | 623.37 | [M+Na]+ | 4 | 10.2 | 0.00957 | L-Serine,2,3-bis[(1-oxododecyl)oxy]propyl hydrogen phosphate(ester) (PS (12:0/12:0)[U]) | 40800 |
| 9 | C_33_H_43_N_7_O_5_ | 617.33 | [M+H]+ | 3 | 9.9 | 0.00779 | Lys Val Trp Trp | 174657 |
| 10 | C_14_H_26_O_4_Si | 286.16 | [M+Na]+ | 0 | 9.3 | 0.00035 | 2,3,4-Trioxycyclopentanone | 69403 |
| High dose PZQ treatment | | | | | | | | |
| 1 | C_38_H_77_NO_5_ | 627.58 | [M+H]+ | 1 | 27.5 | 4.9170e^-6^ | N-(2-hydroxyeicosanoyl)-hydroxysphinganine (Cer (t18:0/20:0(2OH))) | 103031 |
| 2 | C_35_H_68_NO_10_P | 693.45 | [M+H]+ | 2 | 20.7 | 4.5402e^-7^ | 1-dodecanoyl-2-heptadecanoyl-glycero-3-phosphoserine (PS (12:0/17:0)) | 77714 |
| 3 | C_30_H_43_N_7_O_5_ | 581.33 | [M+H]+ | 1 | 16.0 | 2.2878e^-7^ | Phe Phe Ile Arg | 137234 |
| 4 | C_35_H_68_NO_10_P | 693.45 | [M+H]+ | 2 | 15.7 | 1.0877e^-6^ | 1-tridecanoyl-2-hexadecanoyl-glycero-3-phosphoserine (PS (13:0/16:0)) | 77736 |
| 5 | C_19_H_32_O_2_ | 292.24 | [M+H]+ | 1 | 15.6 | 0.00001 | 8-hydroxy-11E-octadecen-9-ynoic acid | 35661 |
| 6 | C_33_H_64_NO_10_P | 665.42 | [M+H]+ | 1 | 15.3 | 2.2593e^-6^ | 1-O-hexadecanoyl-2-O-(9-carboxyoctanoyl)-sn-glyceryl-3-phosphocholine (PAz-PC) | 63020 |
| 7 | C_29_H_56_NO_10_P | 609.36 | [M+H]+ | 1 | 15.2 | 0.00004 | 1-palmitoyl-2-glutaryl phosphatidylcholine (PGPC) | 45374 |
| 8 | C_39_H_77_O_12_P | 768.51 | [M+H]+ | 1 | 15.1 | 1.5017e^-6^ | 1-hexadecyl-2-tetradecanoyl-glycero-3-phospho-(1'-myo-inositol) (PI (O-16:0/14:0)) | 80988 |
| 9 | C_35_H_66_NO_10_P | 691.44 | [M+Na]+ | 0 | 14.9 | 0.00993 | 1-dodecanoyl-2-(9Z-heptadecenoyl)-glycero-3-phosphoserine (PS (12:0/17:1(9Z))) | 77715 |
| 10 | C_36_H_71_N_2_O_7_P | 674.49 | [M+K]+ [M+Na]+ | 1 | 14.6 | 4.7003e^-7^ | N-(2-hydroxy-11Z-eicosenoyl)-tetradecasphing-4-enine-1-phosphoethanolamine (PE-Cer(d14:1(4E)/20:1(11Z)(2OH))) | 103100 |

Note: Highlighted row is the metabolites those were mentioned in main text.
