# Supplementary material for: Metabolomics reveal alterations in arachidonic acid metabolism in Schistosoma mekongi after exposure to praziquantel
Source: PLoS Negl Trop Dis. 2021 Sep 2;15(9):e0009706. doi: 10.1371/journal.pntd.0009706 (PMC8412319; doi:10.1371/journal.pntd.0009706)
Supplement: S2 Table — The pairwise comparison was performed on these results. Anandamide was highly produced in S. mekongi after low-, medium-, and high-dose PZQ treatment. (DOCX) [file pntd.0009706.s008.docx]

**S2 Table. Top-10 metabolites of *S. mekongi* with decreased level after low-, medium-, and high-dose PZQ treatment using pairwise comparisons.**

| **Number** | **Chemical formula** | **Exact mass** | **Ion adduct** | **Mass error (ppm)** | **Fold change** | ***p*-*value*** | **Potential metabolite** | **METLIN ID** |
| --- | --- | --- | --- | --- | --- | --- | --- | --- |
| Low dose PZQ treatment | | | | | | | | |
| 1 | C_34_H_64_O_2_ | 504.49 | [M+H]+ | 4 | -50.2 | 0.00002 | Linoleyl palmitate | 97112 |
| 2 | C_24_H_48_O_2_ | 368.36 | [M+Na]+ | 1 | -38.3 | 0.00031 | 22-methyl-tricosanoic acid | 4300 |
| 3 | C_22_H_36_O_2_ | 332.27 | [M+H]+ | 2 | -33.7 | 2.7551e^-6^ | Arachidonic acid ethyl ester | 404 |
| 4 | C_21_H_33_N_5_O_8_ | 483.23 | [M+H]+ | 0 | -24.8 | 1.1885e^-6^ | Lys Ser Ser Tyr | 173798 |
| 5 | C_22_H_32_O_2_ | 328.24 | [M+H]+ | 1 | -24.4 | 0.01189 | Retinol Acetate | 41508 |
| 6 | C_21_H_31_N_5_O_8_ | 481.21 | [M+Na+NH_3_]+ | 2 | -23.4 | 0.00016 | Asp Gly Lys Tyr | 121659 |
| 7 | C_22_H_35_NO_2_ | 350.30 | [M+H]+ | 1 | -22.6 | 1.1412e^-7^ | Anandamide (20:5, n-3) | 36743 |
| 8 | C_22_H_32_O_2_ | 328.24 | [M+H]+ | 1 | -21.7 | 2.0384e^-6^ | 3a-OH-5aH-Degestrel | 1955 |
| 9 | C_18_H_35_NO | 281.27 | [M+H]+ | 1 | -20.8 | 6.4109e^-7^ | Elaidamide | 36674 |
| 10 | C_24_H_34_N_6_O_10_ | 566.23 | [M+Na]+ | 1 | -19.1 | 0.00032 | Glu Gln Gln Tyr | 132959 |
| Medium dose PZQ treatment | | | | | | | | |
| 1 | C_22_H_36_O_2_ | 332.27 | [M+H]+ | 2 | -53.7 | 9.6766e^-7^ | Arachidonic acid ethyl ester | 404 |
| 2 | C_22_H_32_O_2_ | 328.24 | [M+H]+ | 2 | -49.6 | 7.8177e^-7^ | Retinol Acetate | 41508 |
| 3 | C_14_H_24_O_4_ | 256.16 | [M+H-C_6_H_8_O_6_]+ | 1 | -44.0 | 0.00001 | 5-Nonyltetrahydro-2-oxo-3-furancarboxylic acid | 87337 |
| 4 | C_15_H_20_N_2_S | 260.13 | [M+Na+  HCOOH]+ | 5 | -39.5 | 0.00340 | Methaphenilene | 68854 |
| 5 | C_19_H_28_N_2_O_4_ | 348.20 | [M+Na]+ | 2 | -37.3 | 6.3530e^-7^ | Roxatidine acetate | 85590 |
| 6 | C_34_H_64_O_2_ | 504.49 | [M+H]+ | 4 | -36.3 | 0.00002 | Linoleyl palmitate | 97112 |
| 7 | C_22_H_35_NO_2_ | 350.30 | [M+H]+ | 1 | -33.9 | 8.3850e^-8^ | Anandamide (20:5, n-3) | 36743 |
| 8 | C_37_H_34_O_7_ | 590.23 | [M+Na+NH_3_]+ | 1 | -33.7 | 0.00019 | Triuvaretin | 52278 |
| 9 | C_21_H_33_N_5_O_8_ | 483.23 | [M+H]+ | 0 | -31.2 | 2.8380e^-7^ | Lys Ser Ser Tyr | 173798 |
| 10 | C_20_H_32_O_2_ | 304.24 | [M+H]+ | 3 | -29.2 | 2.4976e^-6^ | 8,11-eicosadiynoic acid | 24087 |
| High dose PZQ treatment | | | | | | | | |
| 1 | C_15_H_20_N_2_S | 260.13 | [M+H]+ [M+2H]_2_+ | 5 | -145.0 | 0.00163 | Methaphenilene | 68854 |
| 2 | C_18_H_35_NO | 281.27 | [M+H]+ | 1 | -18.1 | 6.7926e^-7^ | Elaidamide | 36674 |
| 3 | C_21_H_31_N_5_O_8_ | 481.21 | [M+Na+NH3]+ | 2 | -17.3 | 0.00012 | Ala Gln Thr Tyr | 109019 |
| 4 | C_22_H_32_O_2_ | 328.24 | [M+H]+ | 2 | -16.2 | 2.2603e^-6^ | Retinol Acetate | 41508 |
| 5 | C_36_H_64_O_2_ | 528.49 | [M+H]+ | 1 | -15.5 | 0.00244 | Linolenyl oleate | 97131 |
| 6 | C_22_H_37_NO_2_ | 347.28 | [M+H]+ | 0 | -14.2 | 1.4806e^-11^ | Arachidonoyl Ethanolamide | 7321 |
| 7 | C_22_H_36_O_2_ | 332.27 | [M+H]+ | 1 | -14.0 | 3.2502e^-6^ | Arachidonic acid ethyl ester | 404 |
| 8 | C_36_H_68_O_4_ | 564.51 | [M+H]+ | 3 | -13.8 | 0.0100 | 11-carboxy-1-hexylundecyl ester-9Z-octadecenoic acid | 263595 |
| 9 | C_34_H_68_O_3_ | 524.51 | [M+H-C_3_H_2_O_3_]+ | 2 | -13.8 | 0.00456 | Hydroxyphthioceranic acid | 73734 |
| 10 | C_12_H_24_N_2_O_7_ | 308.15 | [M+H]+ | 1 | -13.7 | 0.00092 | Fructoselysine | 71227 |

Note: Highlighted row is the metabolites those were mentioned in main text.

In high dose group, anandamide was the most increased metabolite number 12^th^, therefore, it did not present in the table.
