# Supplementary material for: Metabolomics reveal alterations in arachidonic acid metabolism in Schistosoma mekongi after exposure to praziquantel
Source: PLoS Negl Trop Dis. 2021 Sep 2;15(9):e0009706. doi: 10.1371/journal.pntd.0009706 (PMC8412319; doi:10.1371/journal.pntd.0009706)
Supplement: S3 Table — Numbers in the table represent percentage identity matrices. Fatty acid amide hydrolases among parasites had high percent similarities. (DOCX) [file pntd.0009706.s009.docx]

**S3 Table**. **Alignment of *Homo sapiens, S. mekongi, S. japonicum, S. mansoni, S. haematobium, S. bovis, Fasciola hepatica, Paragonimus westermani, Clonorchis sinensis,* and *Echinococcus granulosus* fatty acid amide hydrolase sequences**. Numbers in the table represent percentage identity matrices.

|  | *H. sapiens* | *F. hepatica* | *P. westermani* | *C. sinensis* | *E. granulosus* | *S. mansoni* | *S. mekongi* | *S. japonicum* | *S. bovis* | *S. haematobium* |
| --- | --- | --- | --- | --- | --- | --- | --- | --- | --- | --- |
| *H. sapiens* | 100.00 | 34.22 | 35.37 | 34.05 | 33.39 | 31.90 | 31.23 | 30.51 | 30.36 | 29.37 |
| *F. hepatica* | 34.22 | 100.00 | 60.91 | 57.81 | 30.00 | 27.85 | 26.86 | 26.86 | 27.68 | 26.99 |
| *P. westermani* | 35.37 | 60.91 | 100.00 | 66.09 | 28.47 | 27.04 | 27.01 | 27.19 | 26.68 | 25.73 |
| *C. sinensis* | 34.05 | 57.81 | 66.09 | 100.00 | 27.22 | 27.12 | 27.82 | 27.09 | 27.10 | 26.73 |
| *E. granulosus* | 33.39 | 30.00 | 28.47 | 27.22 | 100.00 | 34.14 | 33.85 | 34.60 | 35.94 | 34.94 |
| *S. mansoni* | 31.90 | 27.85 | 27.04 | 27.12 | 34.14 | 100.00 | 41.95 | 42.28 | 40.88 | 38.76 |
| *S. mekongi* | 31.23 | 26.86 | 27.01 | 27.82 | 33.85 | 41.95 | 100.00 | 90.44 | 71.95 | 70.18 |
| *S. japonicum* | 30.51 | 26.86 | 27.19 | 27.09 | 34.60 | 42.28 | 90.44 | 100.00 | 71.04 | 68.52 |
| *S. bovis* | 30.36 | 27.68 | 26.68 | 27.10 | 35.94 | 40.88 | 71.95 | 71.04 | 100.00 | 96.36 |
| *S. haematobium* | 29.37 | 26.99 | 25.73 | 26.73 | 34.94 | 38.76 | 70.18 | 68.52 | 96.36 | 100.00 |
